# Impact of Hydrophobic Chains in Five-Coordinate Glucoconjugate Pt(II) Anticancer Agents

**DOI:** 10.3390/ijms24032369

**Published:** 2023-01-25

**Authors:** Alfonso Annunziata, Paola Imbimbo, Maria Elena Cucciolito, Giarita Ferraro, Vincenzo Langellotti, Alessandra Marano, Massimo Melchiorre, Gabriella Tito, Marco Trifuoggi, Daria Maria Monti, Antonello Merlino, Francesco Ruffo

**Affiliations:** 1 Institute Parisien de Chimie Moléculaire, Campus Pierre et Marie Curie, Sorbonne Université, 4 Place Jussieu, 75005 Paris, France; 2 Dipartimento di Scienze Chimiche, Università degli Studi di Napoli Federico II (Complesso Universitario di Monte S. Angelo), Via Cintia 21, 80126 Napoli, Italy; 3 Consorzio Interuniversitario di Reattività Chimica e Catalisi (CIRCC), Via Celso Ulpiani 27, 70126 Bari, Italy

**Keywords:** platinum(II), five-coordinate complexes, glycoconjugation, cytotoxic activity, DNA binding, protein X-ray structure, lipophilicity

## Abstract

This study describes new platinum(II) cationic five-coordinate complexes (**1-R,R’**) of the formula [PtR(NHC)(dmphen)(ethene)]CF_3_SO_3_ (dmphen = 2,9-dimethyl-1,10-phenanthroline), containing in their axial positions an alkyl group R (methyl or octyl) and an imidazole-based NHC-carbene ligand with a substituent R’ of variable length (methyl or octyl) on one nitrogen atom. The Pt–carbene bond is stable both in DMSO and in aqueous solvents. In DMSO, a gradual substitution of dmphen and ethene is observed, with the formation of a square planar solvated species. Octanol/water partitioning studies have revealed the order of hydrophobicity of the complexes (**1-Oct,Me** > **1-Oct,Oct** > **1-Me,Oct** > **1-Me,Me**). Their biological activity was investigated against two pairs of cancer and non-cancer cell lines. The tested drugs were internalized in cancer cells and able to activate the apoptotic pathway. The reactivity of **1-Me,Me** with DNA and protein model systems was also studied using UV–vis absorption spectroscopy, fluorescence, and X-ray crystallography. The compound binds DNA and interacts in various ways with the model protein lysozyme. Remarkably, structural data revealed that the complex can bind lysozyme via non-covalent interactions, retaining its five-coordinate geometry.

## 1. Introduction

Cisplatin [1] and its derivatives are successfully used in the clinical treatment of cancer diseases. However, their administration is often accompanied by side effects that seriously affect the quality of life of patients [2]. Current strategies to enhance their performance involve the accurate tailoring of the coordination environment of the compounds, to make their action selective towards target cells, improve their stability, facilitate transport in physiological fluids, increase internalization in cells, and favour accumulation in selected biological compartments. This variety of options has produced a plethora of platinum(II) and platinum(IV) complexes, respectively, square planar and octahedral, endowed with biologically active fragments [3,4], modular and stable ligands [5,6,7,8,9,10], or functions that can be interfaced with supramolecular structures [11].

**Figure 1:**
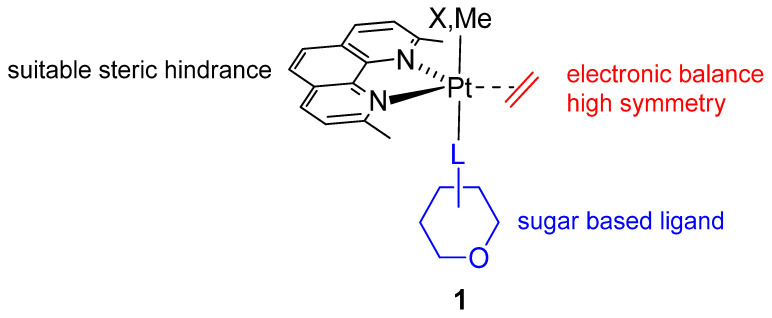
General structure of type **1** complexes.

Recently, our research group has contributed to this emerging area by proposing new five-coordinate platinum(II) glycoconjugate complexes [12,13,14,15] (**1**) with the following structural motifs (Figure 1):

We designed our structures aiming at conferring specific qualities to these agents, taking full advantage of the high versatility of the trigonal bipyramidal (*tbp*) scaffold. This coordinated saturation enhances their stability in biological milieux and hence, their chance at reaching the cellular target intact. This property is guaranteed by the simultaneous presence of the sterically hindered 2,9-dimethyl-1,10-phenanthroline (dmphen) and the π-acid ligand ethene, which balance the delicate electronic equilibrium within the equatorial plane of the trigonal bipyramid [16]. One axial position is occupied by a targeting ligand containing a sugar fragment. Glycoconjugation aims to selectively deliver the agent to cancer cells [17], exploiting the overexpression of glycosyl receptors, to satisfy their increased uptake of carbohydrates (“Warburg effect”), which is necessary to sustain cell growth and proliferation. Remarkable results were obtained with a *tbp* Pt(II) complex containing a glucoconjugate *N*-heterocyclic carbene ligand, which was demonstrated to be highly active and by far more selective for cancer cells, compared to cisplatin [14]. Carbene ligands are highly versatile synthetic platforms that can be variously decorated and confer good resistance to their complexes under hydrolytic conditions such as those of biological fluids [6].

**Figure 2:**
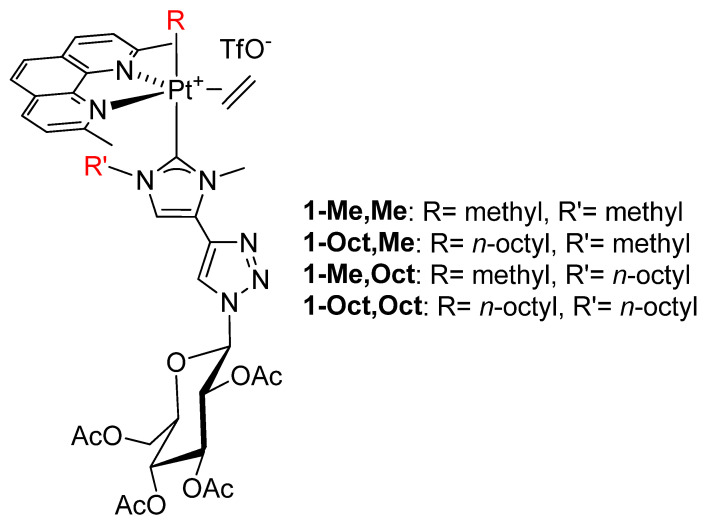
Structure of the complexes prepared within this study.

The other apical position of the *tbp*, which in our previous structures hosted a halide (X) or a methyl (Me), can be further exploited to provide additional features to the complexes. This consideration inspired the possibility of adding further diversity, taking a cue from recent studies on the fine engineering of platinum agents. It has been demonstrated that the introduction of hydrophobic groups in the coordination sphere of metal complexes can have a positive impact on their cytotoxicity, cellular uptake, and selective transport in cellular targets [11,18,19,20,21,22,23,24,25,26]. This work aimed to verify the effect of fatty alkyl chains in the following family of type **1** complexes (Figure 2).

Hydrophobic *n*-octyl groups were added both in the apical position and in the carbene ligand, along with a simple methyl group. Thus, a set of four complexes spanning from the less aliphatic **1-Me,Me** to the more aliphatic **1-Oct,Oct** were synthesised and fully characterized. The ability of **1-Me,Me** to interact with model DNA and protein systems was demonstrated, and studies of cytotoxicity have disclosed that increasing the length of the chain enhances its antiproliferative activity.

## 2. Results and Discussion

### 2.1. Synthesis and Characterization

**Scheme 1:**
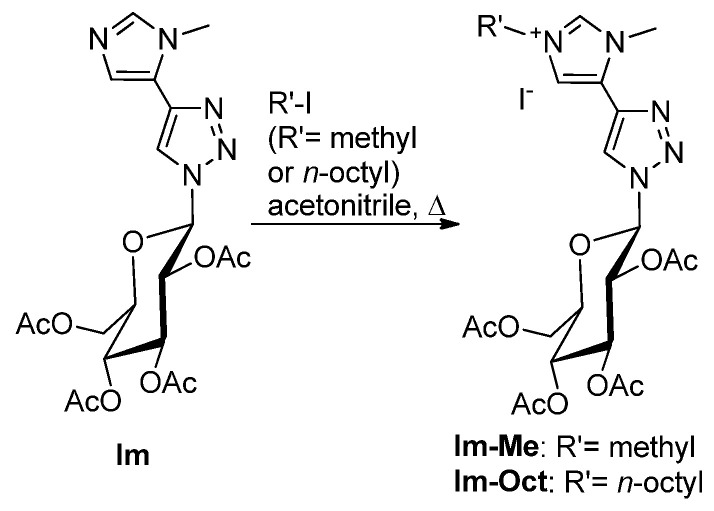
Synthesis of the pro-carbene species **Im-Me** and **Im-Oct**.

The synthesis of the target platinum(II) complexes involved the initial preparation of pro-carbene imidazolinium salts **Im-R** (Scheme 1).

**Figure 3:**
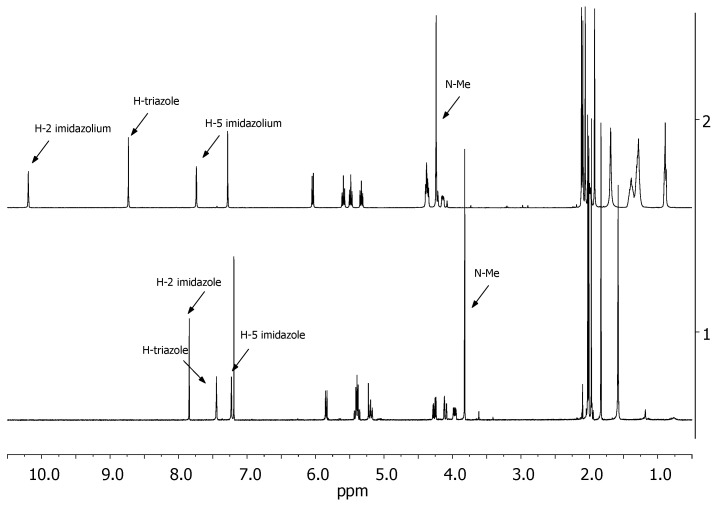
The ^1^H NMR spectra of **Im** (trace 1) and **Im-Oct** (trace 2) in CDCl_3_.

The imidazole precursor **Im** [12] was treated with an excess of the appropriate alkyl iodide to yield the pro-carbene precursor **Im-Me** or **Im-Oct** (Scheme 1). The alkylation was carried out in refluxing acetonitrile and the cationic products were isolated in a nearly quantitative yield by the removal of the solvent. A high-frequency shift of the NMR signals (Figures S1 and S2) of the three heterocyclic protons is indicative of the presence of a positive charge. For instance, Figure 3 compares the NMR spectra of **Im** and **Im-Oct** in deuterochloroform.

**Scheme 2:**
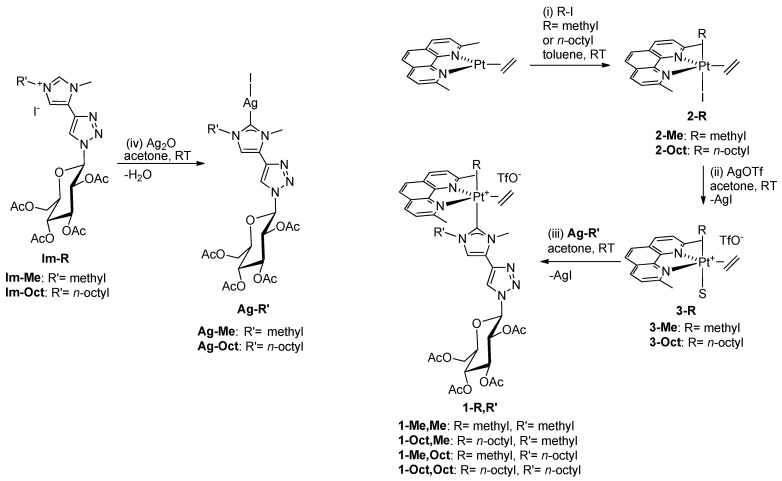
Synthesis of the silver precursors (**Ag-R’**) and platinum **1-R,R’** complexes.

The platinum precursors used to synthesise the glucoconjugated complexes were obtained through the oxidative addition (path i of Scheme 2) of methyl iodide or *n*-octyl iodide to the Pt(0) precursor [Pt(dmphen)(ethene)], affording the five-coordinate species **2-Me** and **2-Oct** in excellent yields as light yellow powders. Treatment with silver triflate in acetone promoted the formation of the solvato species **3-Me** and **3-Oct**. They were immediately reacted with the appropriate silver carbene complex **Ag-R’** (iii), obtained by treating the imidazolinium salts **Im-R’** with silver oxide (iv). Transfer of the carbene ligand, assisted by the precipitation of Ag-I, completed the synthesis of the type **1-R,R’** complexes.

**Figure 4:**
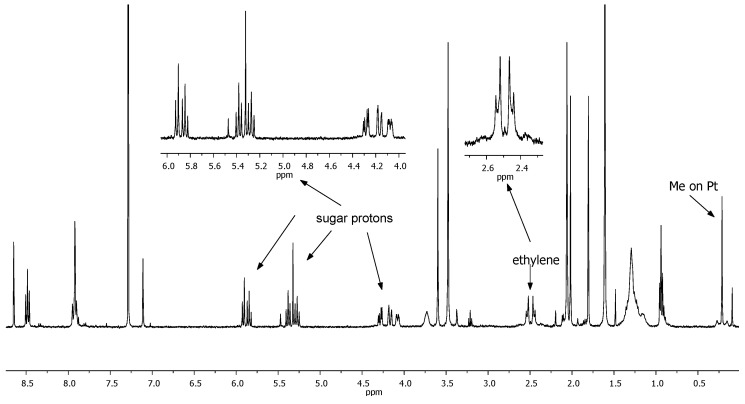
The ^1^H NMR spectrum of **1-Me,Oct** in CDCl_3_.

The products (complete names in Table S1) were characterized by ^1^H and ^13^C NMR spectroscopy (Figures S3–S12), which allowed for the unequivocal assessment of their structure, thanks to the following relevant observations. The two halves of dmphen are not equivalent due to the presence of the chiral sugar ligand. The four ethene protons resonate as an AA’BB’ multiplet or accidentally as a singlet flanked by the expected satellites due to coupling with ^195^Pt nuclei. The chemical shift was at a low frequency (2–2.5 ppm) due to the intense π-backdonation in the Pt–alkene bond. In accordance with this, olefin carbon signals were also detected at ca. 30 ppm. The signal of the methyl on platinum was at a low frequency in both the proton and carbon spectra, while the *n*-octyl chain gave rise to a crowded set of signals in the aliphatic region. Sugar protons showed the expected pattern of signals with H,H-couplings in accordance with their relative position in the glucose ring. Finally, as expected, the C(carbene)–Pt signal was found at a high frequency (173.8–172 ppm). Figure 4 displays the proton NMR spectrum of **1-Me,Oct**, in which some of the above evidence has been highlighted.

### 2.2. In-Solution Stability of **1-R,R’** Complexes

The stability of the complexes in DMSO-d_6_, the solvent used to prepare stock solutions for biological experiments, was evaluated by recording their UV–vis absorptions and ^1^H-NMR spectra over time.

**Figure 5:**
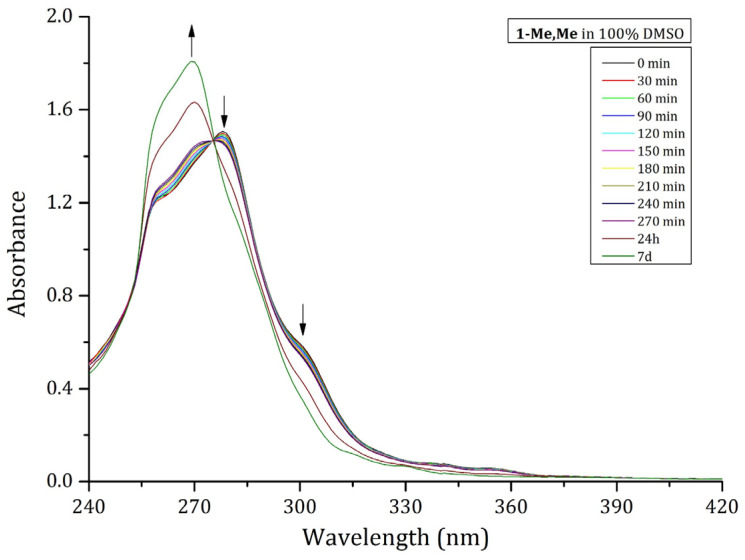
Time-course UV–vis spectra of 50 μM of **1-Me,Me** in DMSO.

**Scheme 3:**
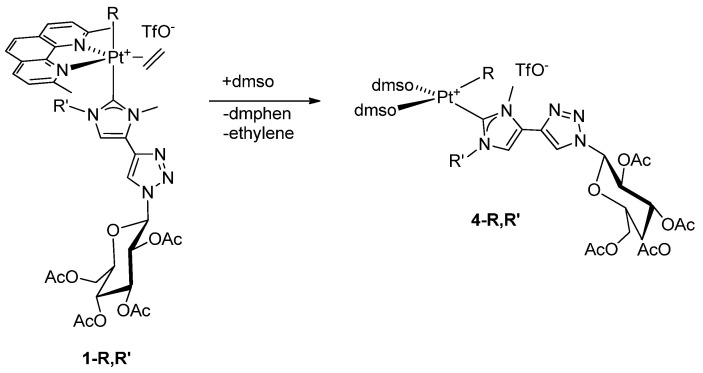
Reactivity of **1-R,R’** complexes in DMSO-d_6_.

UV–vis absorption spectra of **1-Me,Me** were recorded in DMSO for 5 h and then after 24 h and 7 days (Figure 5). The Pt complex showed a maximum of absorbance at 278 nm and two shoulders at 260 and 300 nm. The kinetic measurements highlight the poor stability of **1-Me,Me** in DMSO since, after just a few minutes, a variation in the spectral profile occurred. In fact, the λ_max_ experienced a blue shift from 278 to 269 nm, giving rise to an isosbestic point at 275 nm. In addition, a decrease in the absorbance of the shoulder at 300 nm is observed, together with an absorbance increase in the shoulder at 260 nm. These findings suggest that an exchange of metal ligands with DMSO occurs. The exchange continued over time and the spectra significantly changed from 0 to 7 days. It is highly probable that a slow release of ethene and dmphen can occur. This would lead to the formation of the square planar species of type **4-R,R’**, as reported in Scheme 3.

**Figure 6:**
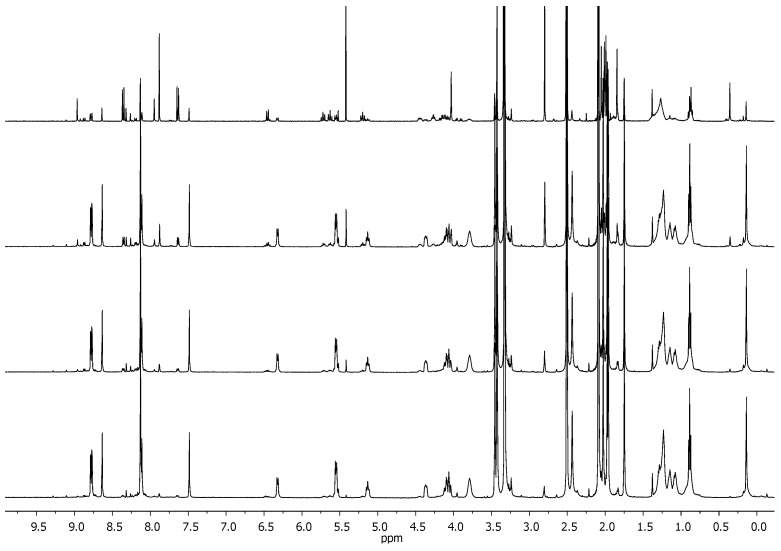
The ^1^H NMR spectrum of **1-Me,Oct** in DMSO-d_6_ after dissolution (bottom), 1.5, 5, and 24 h (up).

The NMR data confirm (Figures S13–S16) this hypothesis and demonstrate that the progress of the process is influenced by the steric hindrance of the alkyl substituent on Pt: after 24 h, the molar ratios of **1-R,R’**/**4-R,R’** are, respectively, 1.8/1 for **1-Me,Me** and **1-Me,Oct** and 4.5/1 for **1-Oct,Me** and **1-Oct,Oct**. This finding indicates that the presence of the octyl chain on the platinum centre represents the major obstacle to exchange. In all cases, no cleavage of the Pt–carbene bond was detected. Figure 6 reports the ^1^H NMR spectra of **1-Me,Oct** after dissolution, 1.5, 5, and 24 h.

Attempts to evaluate their stability in aqueous solutions were complicated due the poor solubility of the complexes in water. Only for **1-Me,Me** was it possible to prepare a solution in the mixed solvent D_2_O:DMSO-d_6_ 10:1. After 24 h, no appreciable changes in the NMR spectra of the complex were observed (Figure S17). To further study the stability of **1-Me,Me** in aqueous solvents, UV–vis absorption spectra over time were collected under the two experimental conditions (20% ethylene glycol, 0.1 M sodium acetate buffer at pH 4.0 and 0.6 M sodium nitrate and 2.0 M sodium formate, 0.1 M Hepes buffer at pH 7.5), used to obtain crystals of the adducts with the model protein hen egg white lysozyme (HEWL). HEWL has been used as a model system to study the interaction with proteins of several metallodrugs [27,28,29,30], including cisplatin [31,32,33], oxaliplatin [34,35], and carboplatin [31,32]. The **1-Me,Me** complex appeared more stable in these solutions than in DMSO (Figure S18A,B). Under the first condition, **1-Me,Me** showed three signals: a maximum at 275 nm and two minor peaks at 255 and 313 nm (Figure S18A). Over time, a red shift of the λ_max_ from 275 to 278 nm and of the peak at 313 nm, which shifted up to 317 nm, was observed. Under the other condition, spectra of **1-Me,Me** showed a λ_max_ at 277 nm and a minor peak at 300 nm (Figure S18B). Under this condition, the compound was stable over time and only a slight precipitation of the sample took place. Such results convinced us that five-coordinate compounds are the species administered to cells if DMSO stock solutions are immediately diluted in an aqueous medium.

### 2.3. Partition Coefficients of **1-R,R’** Complexes

The partition coefficients in octanol/water (log P_o/w_) were measured using the shake-flask method [36], by calculating the equilibrium concentrations through UV spectra (Table 1).

Complexes with the methyl group on the platinum centre are more hydrophilic than the ones with *n*-octyl. Among this first type of complexes, an increase in the length of its R group is connected to an increase in its hydrophobicity. Conversely, for complexes with *n*-octyl on platinum, there is an inversion of this trend. Therefore, the order of hydrophobicity of the complexes is the following: **1-Oct,Me** > **1-Oct,Oct** > **1-Me,Oct** > **1-Me,Me**. This tendency, although counterintuitive because it does not reflect the content of the carbon atoms in the complexes, has already been observed in other studies on platinum agents and has been attributed to the extent of ligand surface exposition [37,38].

### 2.4. Interaction with DNA

**Figure 7:**
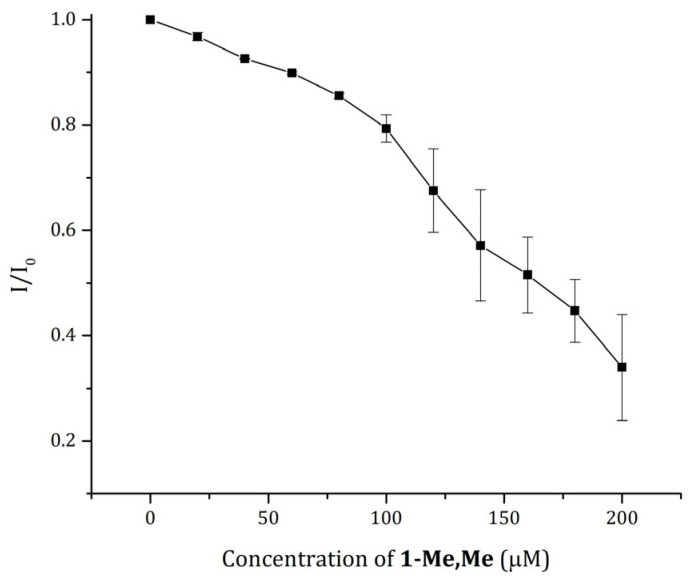
Fluorescence quenching of a 20 μM EB-DNA complex upon titration with a solution of **1-Me,Me** in 0.05 M of ammonium acetate at a pH of 7.5, reported as a function of the Pt compound concentration.

The interaction of Pt-based drugs with biological macromolecules deeply affects their biological activity. DNA represents a target for this kind of metallodrug, while proteins are considered both as carriers and targets. To obtain information on the reactivity of the synthesised Pt compounds with DNA, fluorescence intercalation displacement assays [39] have been performed using ethidium bromide (EB) as the DNA intercalator. When bound to DNA, EB has an emission fluorescence of significant intensity. On the other hand, when it is displaced by a competitive DNA-binding molecule, it undergoes quenching by water molecules. Thus, if a molecule binds DNA by displacing EB, a significant reduction in the fluorescence intensity of the EB-DNA complex will be observed. Upon addition of **1-Me,Me** to the EB-DNA complex, a significant reduction in fluorescence intensity was found (Figure 7). This finding demonstrates that the complex binds DNA.

### 2.5. Interaction with Proteins

To obtain information on the reactivity of **1-Me,Me** with proteins, the X-ray structure of the adducts that the Pt compound forms with HEWL was solved. It was preventively verified that under these conditions, the complex does not degrade in the presence of the protein (Figure S18C,D).

**Figure 8:**
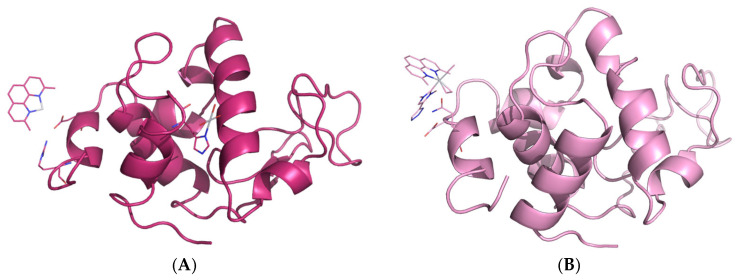
Overall structures of the adducts formed upon the reaction of **1-Me,Me** with HEWL in 20% ethylene glycol, 0.1 M sodium acetate buffer at pH 4.0, and 0.6 M sodium nitrate (Structure **A**) and in 2.0 M sodium formate and 0.1 M Hepes at pH 7.5 (Structure **B**).

The structures were obtained using crystals grown in 20% ethylene glycol, 0.1 M sodium acetate buffer at pH 4.0, and 0.6 M sodium nitrate (Structure **A**, Figure 8A) and in 2.0 M sodium formate and 0.1 M Hepes at pH 7.5 (Structure **B**, Figure 8B), refined at 1.25 and 1.33 Å resolutions, respectively (Table S2).

In both cases, the Pt compound binding did not significantly affect the protein’s tertiary structure. The root mean square deviation of the Cα atoms from the structure of the metal-free HEWL (PDB code 193L) [40] were within the range of 0.16–0.23 Å. However, under the two conditions, different results were obtained.

**Figure 9:**
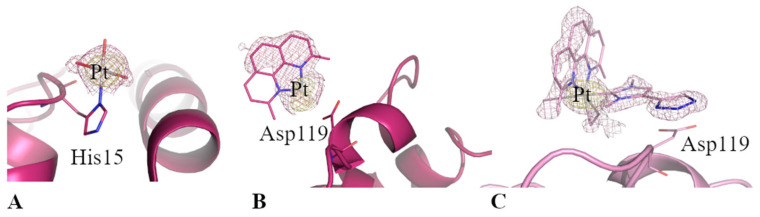
Pt binding sites in Structures **A** (panels (**A**,**B**)) and **B** (panel (**C**)). Atoms were placed where we were confident of their assignment. The 2Fo-Fc maps (violet) are shown at the 1.0 σ values. An anomalous difference electron density map (yellow) is shown at the 3σ contour level. The Pt atoms are shown in grey.

In Structure **A**, two Pt binding sites were observed (Figure 9A,B), close to the His15 and Asp119 side chains. In both these binding sites, Pt seems to adopt a square planar geometry, suggesting that the complex lost its 5C geometry. The definition of Pt ligands at these metal binding sites is not clear. In the final Structure **A**, at the His15 binding site, a Pt atom coordinated by water molecules was modelled, while close to Asp119, it appeared that a dmphen ligand could be present. Both His15 and Asp119 have already been identified as Pt binding sites [34,35,41,42]. In Structure **B**, a single Pt binding site was observed (Figure 9C). Notably, in this structure, a Pt-containing fragment non-covalently bound to the protein was found. This Pt-containing fragment retained its 5C geometry. In fact, the electron density map allowed for modelling all the ligands of the platinum centre, if the glucoconjugated part of the carbene ligand was excluded (Figure 9C). This finding agrees with the results of UV–vis absorption spectroscopy suggesting that **1-Me,Me** does not show any variation in its structure in the presence of the protein, under the crystallization conditions used to obtain Structure **B**.

### 2.6. Effects of Complexes on Cell Viability

To evaluate their biological activity, the effects of the four complexes on cell viability were evaluated in eukaryotic cell models. For this purpose, experiments were performed on two cancer cell lines, i.e., human epidermoid carcinoma cells (A431) and murine fibroblast BALB/c-3T3 transformed with the SV40 virus (SVT2), and on two non-cancer cell lines, i.e., immortalized human keratinocytes (HaCaT) and immortalized murine fibroblasts (BALB/c-3T3). Cells were incubated with increasing concentrations of **1,Me-Me**, **1,Oct-Me**, **1,Me-Oct**, and **1,Oct-Oct** (from 0.1 to 50 μM). After 48 h of incubation, cytotoxicity was determined by MTT assay [43] and cell survival was expressed as the percentage of viable cells in the presence of each complex compared with that of the untreated cells (Figure S19).

The IC_50_ values (the concentration of the complex able to reduce cell viability to 50%) and the selectivity indexes (SI) (the ratio between the IC_50_ values of the non-cancer cell line and cancer cells) of the compounds are reported in Table 2.

Overall, despite the IC_50_ values being in the low micromolar range, only a slight selectivity towards cancer cells was observed. Interestingly, in the case of the A431 cells, the IC_50_ values increased with hydrophilicity, with **1-Oct,Me** being the most active and **1-Me,Me** being the least. Moreover, the SI decreased with hydrophilicity, suggesting that the higher the hydrophobicity of the drug, the higher the selectivity observed. Indeed, in the case of the A431/HaCaT couple, it was pleasantly observed that greater activity of **1-Oct,Me** was accompanied by a higher SI (2.78). By contrast, in the case of SVT2, the highest SI was found for the most hydrophilic compound (**1-Me,Me,** SI of 2.65) with respect to **1-Oct,Me**, which showed the lowest SI value.

### 2.7. Cytotoxicity Pathways of **1-Me,Me** and **1-Oct,Me**

**Figure 10:**
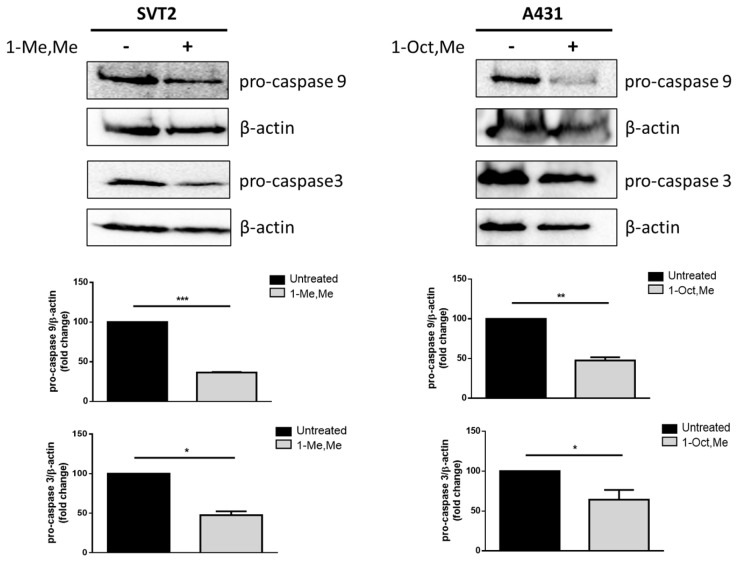
Apoptosis analysis in cancer cell lines. Cells were incubated with the molecule being tested, with each one at its IC_50_ value (9.8 μM **1-Me,Me** on SVT2 and 2.3 μM **1-Oct,Me** on A431). After 48 h, protein extracts were analysed by Western blot using anti-pro-caspases 9 and 3. β-actin was used as the loading control. The densitometric analysis is reported below the Western blot images. Black bars, untreated cells; grey bars, treated cells. Data shown are the means ± S.D. of three independent experiments. * indicates *p* < 0.05; ** indicates *p* < 0.01; *** indicates *p* < 0.001 with respect to untreated cells.

To further investigate the molecular mechanisms of cell death induced by the two complexes that showed the highest SI values, the effects of **1-Me,Me** and **1-Oct,Me** were analysed in cancer cells. In particular, SVT2 and A431 were incubated for 48 h with **1-Me,Me** and **1-Oct,Me**, using the IC_50_ value concentrations. At the end of incubation, uptake and cell death mechanisms were evaluated. As shown in Table 3, both complexes were able to enter cells, but **1-Oct,Me** was more internalized on A431 with respect to **1-Me,Me** on SVT2. This result is in agreement with the higher toxicity of **1-Oct,Me**, as it is better able to enter cells at a lower concentration. Finally, apoptosis was investigated by Western blot analyses using specific antibodies against pro-caspases 9 and 3. As shown in Figure 10, both complexes were able to induce the activation of apoptosis, as a significant decrease in pro-caspases 9 and 3 levels was observed in both cell lines.

## 3. Materials and Methods

**Figure 11:**
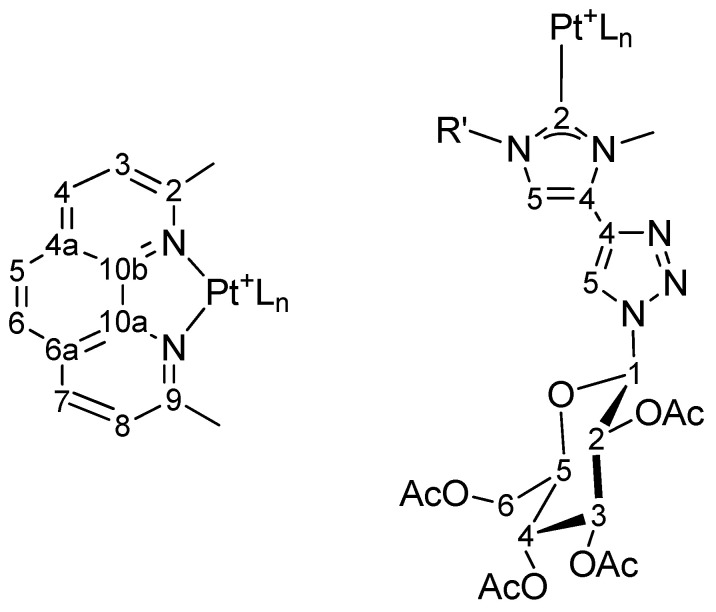
Numbering scheme for the carbon atoms within the rings.

All solvents and reagents were purchased from Merck KGaA (Darmstadt, Germany) and used without any further purification. NMR spectra were recorded using a 400 Bruker Avance with Ultrashield or a 500 Varian Inova spectrometer at 298 K. Chemical shifts are given in parts per million (ppm, *δ*), referenced to the solvent peaks of CDCl_3_ (^1^H NMR *δ* = 7.26, ^13^C NMR *δ* = 77), DMSO-d_6_ (^1^H NMR *δ* = 2.50, ^13^C NMR *δ* = 39.52), and D_2_O (^1^H NMR *δ* = 4.79 ppm). Coupling constants are quoted in Hz (J). The ^1^H NMR and ^13^C NMR splitting patterns were designated as singlet (s), doublet (d), triplet (t), quartet (q), double doublet (dd), and broad (br). Splitting patterns with a difficult interpretation or visualization were designated as multiplet (m). The compounds **Im-Me** [44], [Pt(dmphen)(ethene)] [45], and **2-Me** [44] were prepared according to procedures in the literature. In describing the NMR assignments, the scheme adopted for numbering the carbon atoms within the rings is reported as the following (Figure 11).

For the ICP-MS analyses, high-purity water (resistivity of 18.2 MΩ cm) was obtained from a Milli-Q unit (Millipore, Burlington, MA, USA) and was used for the standard solution preparation and sample dilutions. Nitric acid (HNO_3_, 69% *v*/*v* Ultratrace^@^ ppb-trace analysis grade) was provided by Scharlab (Barcelona, Spain). A certified reference solution containing Pt at 100 mg/L of ultrapure grade for ICP by VWR Avantor^®^ (Radnor Township, PA, USA) was used.

### 3.1. Synthesis of **Im-Oct**

*n*-Octyl iodide (0.85 mL, 4.7 mmol) was added to a solution of **Im** (0.15 g, 0.31 mmol) in 2 mL of acetonitrile. The light yellow solution was stirred under reflux for 3 h. The solvent was removed and the orange oil was washed with *n*-hexane. A white powder was obtained (yield: 97%). ^1^H NMR (500 MHz, 298 K, CDCl_3_): *δ* 10.17 (s, 1H, H2 imidazolium), 8.71 (s, 1H, H5 triazole), 7.72 (s, 1H, H5 imidazolium), 6.02 (d, 1H, ^3^*J*_H1–H2_ = 9.3 Hz, H1 glu), 5.57 (t, 1H, ^3^*J*_H2–H3_ = 9.4 Hz, H2 glu), 5.46 (t, 1H, ^3^*J*_H3–H4_ = 9.5 Hz, H3 glu), 5.31 (t, 1H, ^3^*J*_H4–H5_ = 9.8 Hz, H4 glu), 4.41–4.30 (m, 3H, H6 glu, and NCH_2_), 4.22 (s, 3H, nMe), 4.19 (dd, 1H, ^2^*J*_H6–H6′_ overlapped, ^3^*J*_H5–H6_ = 1.8 Hz, H6′ glu), 4.15–4.08 (m, 1H, H5 glu), 2.09 (s, 3H, OAc), 2.07 (s, 3H, OAc), 2.04 (s, 3H, OAc), 2.02–1.93 (m, 2H, CH_2_), 1.90 (s, 3H, OAc), 1.47–1.17 (m, 10H, CH_2_), and 0.95–0.77 (m, 3H, Me). ^13^C NMR (125 MHz, 298 K, CDCl_3_): *δ* 170.6 (CO), 169.9 (CO), 169.4 (CO), 169.0 (CO), 137.7 (C2 imidazole), 134.1 (C4 imidazole), 126.4 (C4 triazole), 124.0 (C5 triazole), 120.2 (C5 imidazole), 85.8 (C1 glu), 75.3, 72.6, 70.5, and 67.6 (C2–C5 glu), 61.5 (C6 glu), 50.7 (NCH_2_), 36.7 (nMe), 31.7, 30.2, 29.1, 29.0, 26.3, and 22.6 (6 CH_2_), 20.9 (OAc), 20.6 (x2, OAc), 20.4 (OAc), and 14.1 (Me).

### 3.2. Synthesis of **2-Oct**

*n*-Octyl iodide (0.5 mL, 2.7 mmol) was added to a suspension of [Pt(dmphen)(ethene)] (0.43 g, 1.0 mmol) in 5 mL of dry toluene and the mixture was stirred at room temperature. After 1 h, *n*-hexane was added to complete the precipitation of a yellow solid, which was recovered and washed with *n*-hexane. The solid was dissolved in dichloromethane, and the solution was filtered on a thin pad of FLORISIL^®^ (100–200 mesh) and crystallized with *n*-hexane (light brown solid—yield: 65%). ^1^H NMR (500 MHz, 298 K, CDCl_3_): *δ* 8.31 (d, 2H, *J* = 8.2 Hz, H4 and H7 dmphen), 7.85 (s, 2H, H5 and H6 dmphen), 7.77 (d, 2H, *J* = 8.2 Hz, H3 and H8 dmphen), 3.33 (s, 6H, Me dmphen), 3.31 (dd, 2H, *J_Pt_* = 85.2 Hz, H ethene—partially overlapped), 2.22 (dd, 2H, *J_Pt_* = 62.0 Hz, ethene), 1.14–0.7 (m, 12H, H_aliphatic_), 0.75 (t, 3H, *J* = 7.3 Hz, Me aliphatic), and 0.36–0.23 (m, 2H). ^13^C NMR (125.7 MHz, 298 K, CDCl_3_): *δ* 161.2 (x2, C2 and C9 dmphen), 145.2 (x2, C10a and C10b dmphen), 137.3 (x2, C4 and C7 dmphen), 128.5 (x2, C4a and C6a dmphen), 126.1 (x2, C5 and C6 dmphen), 125.8 (x2, C3 and C8 dmphen), 31.7, 31.0 (x2), 30.7 and 30.3 (5 CH_2_), 29.2 (x2, Me dmphen), 28.9 (x2) (ethene, *J*_Pt_ = 382 Hz), 22.6 (CH_2_), 19.6 (PtCH_2_, *J*_Pt_ = 638 Hz), and 14.1 (Me).

### 3.3. Synthesis of **Ag-R’** Complexes

The appropriate **Im-R’** precursor (0.30 mmol) was dissolved in 6 mL of acetone. Then, silver(I) oxide (0.046 g, 0.20 mmol) was added. The colourless mixture was stirred for 2 h in the dark. The light yellow mixture was filtered on a Celite^®^ to remove the excess of silver oxide and the resulting colourless solution was used in situ as described in the following paragraph.

### 3.4. Synthesis of **1-R,R’** Complexes

The appropriate type **2-R** complex (0.30 mmol) was suspended in 6 mL of acetone. Then, thallium triflate (0.10 g, 0.30 mmol) was added and the yellow mixture was stirred for 15 min. The suspension was centrifuged to separate the thallium chloride. The resulting solution, containing the type **3-R** complex, was treated with the solution of **Ag-R’** prepared as described above. The mixture was stirred for 60 h in the dark. The resulting solution was filtered on a Celite^®^ to separate the silver iodide. The solvent was removed and a solid was obtained. The crude residue was dissolved in dichloromethane and filtered on a pad of basic activated aluminium oxide. The solution was treated with diethyl ether until the precipitation of a solid and subsequently washed again with diethyl ether. The products were obtained as yellow/light brown solids. **1-Me,Me** (yellow solid—yield: 55%)**.** ^1^H NMR (500 MHz, 298 K, CDCl_3_): *δ* 8.49 (s, 1H, H5 triazole), 8.47 (d, ^3^*J*_H4–H5 or H7–H6_ = 8.1, 1H, H4 or H7 dmphen), 8.45 (d, ^3^*J*_H7–H8 or H4–H5_ = 8.1, 1H, H7 or H4 dmphen), 7.94–7.86 (m, 4H, H3, H5, H6, H8 dmphen), 7.08 (s, 1H, H5 imidazole), 5.85 (d, 1H, ^3^*J*_H1–H2_ = 9.4 Hz, H1 glu), 5.75 (t, 1H, ^3^*J*_H2–H3_ = 9.4 Hz, H2 glu), 5.35 (t, 1H, ^3^*J*_H3–H4_ = 9.4 Hz, H3 glu), 5.24 (t, 1H, ^3^*J*_H4–H5_ = 9.8 Hz, H4 glu), 4.25 (dd, 1H, ^2^*J*_H6–H6′_ = 12.6 Hz, ^3^*J*_H5–H6_ = 4.6 Hz, H6 glu), 4.13 (dd, 1H, ^2^*J*_H6′–H6_ = 12.6 Hz, ^3^*J*_H5–H6′_ = 1.9 Hz, H6′ glu), 4.05–4.00 (m, 1H, H5 glu), 3.56 (s, 3H, N-Me), 3.45 (s, 6H, Me dmphen), 3.33 (s, 3H, N-Me), 2.48 (AA’BB’, 4 H, *J*_Pt_ = 68.4 Hz, ethene), 2.03 (s, 6H, OAc), 1.99 (s, 3H, OAc), 1.78 (s, 3H, OAc), and 0.18 (s, 3H, *J*_Pt_ = 45.7 Hz, PtMe). ^13^C NMR (100 MHz, 298 K, CDCl_3_): δ 172.1 (C carbene), 170.7 (CO), 170.1 (CO), 169.4 (CO), 168.8 (CO), 161.3 and 161.2 (C2 and C9 dmphen), 145.9 (x2, C10a and C10b dmphen), 138.9 and 138.8 (C4 and C7 dmphen), 135.9 (C4 imidazole), 128.7 (x2, C4a and C6a dmphen), 126.7 and 126.6 (C5 and C6 dmphen), 126.1 and 126.0 (C3 and C8 dmphen), 125.6 (C4 triazole), 124.5 and 122.0 (C5 triazole and C5 imidazole), 121.0 (q, CF_3_, *J*_C–F_ = 321 Hz), 85.1 (C1 glu), 74.8, 73.4, 70.0 and 67.7 (C2–C5 of glu), 61.5 (C6 of glu), 37.2 (nMe), 36.4 (nMe), 30.1 (x2, ethene, *J*_Pt_ = 355 Hz), 28.7 (x2, Me dmphen), 20.8 (OAc), 20.7 (x2, OAc), 20.3 (OAc), and -6.6 (PtMe, *J*_Pt_ = 464 Hz). Calculated for C_39_H_46_F_3_N_7_O_12_PtS: C, 43.01; H, 4.26; and N, 9.00. Found: C, 43.28; H, 4.33; and N, 8.87. **1-Me,Oct** (yellow solid—yield: 63%). ^1^H NMR (400 MHz, 298 K, CDCl_3_): *δ* 8.62 (s, 1H, H5 triazole), 8.47 (d, ^3^*J*_H4–H5 or H7–H6_ = 8.7, 1H, H4 or H7 dmphen), 8.45 (d, 1H, ^3^*J*_H7–H8 or H4–H5_ = 8.7, H7 or H4 dmphen), 7.96–7.82 (m, 4H, H3, H5, H6, H8 dmphen), 7.09 (s, 1H, H5 imidazole), 5.89 (d, 1H, ^3^*J*_H1–H2_ = 9.4 Hz, H1 glu), 5.82 (t, 1H, ^3^*J*_H2–H3_ = 9.2 Hz, H2 glu), 5.36 (t, 1H, ^3^*J*_H3–H4_ = 9.2 Hz, H3 glu), 5.25 (t, 1H, ^3^*J*_H4–H5_ = 9.7 Hz, H4 glu), 4.26 (dd, 1H, ^2^*J*_H6–H6′_ = 12.7 Hz, ^3^*J*_H5–H6_ = 4.5 Hz, H6 glu), 4.14 (dd, 1H, ^2^*J*_H6′–H6_ = 12.7, ^3^*J*_H5–H6′_ = 2.3 Hz, H6′ glu), 4.09–4.01 (m, 1H, H5 glu), 3.70 (br, 2H, NCH_2_), 3.57 (s, 3H, nMe), 3.45 (s, 6H, Me dmphen), 2.47 (AA’BB’, 4 H, *J*_Pt_ = 68.4 Hz, ethene), 2.04 (s, 3H, OAc), 2.03 (s, 3H, OAc), 1.99 (s, 3H, OAc), 1.78 (s, 3H, OAc), 1.40–1.10 (m, 12H, CH_2_), 0.92 (t, 3H, Me), and 0.19 (s, 3H, *J*_Pt_ = 46.5 Hz, PtMe). ^13^C NMR (100 MHz, 298 K, CDCl_3_): *δ* 172.0 (C carbene), 170.7 (CO), 170.1 (CO), 169.4 (CO), 168.7 (CO), 161.1 and 161.0 (C2 and C9 dmphen), 145.9 (x2, C10a and C10b dmphen), 138.8 and 138.7 (C4 and C7 dmphen), 135.8 (C4 imidazole), 128.7 and 128.6 (C4a and C6a dmphen), 126.7 and 126.5 (C5 and C6 dmphen), 126.4 and 126.2 (C3 and C8 dmphen), 126.1 (C4 triazole), 124.7 (C5 triazole), 121.0 (q, CF_3_, *J*_C–F_ = 321 Hz), 118.9 (C5 imidazole), 85.0 (C1 glu), 74.7, 73.4, 69.9 and 67.6 (C2–C5 glu), 61.5 (C6 glu), 49.5 (NCH_2_), 36.4 (nMe), 31.8 (CH_2_), 30.6 (CH_2_), 30.1 and 30.0 (ethene), 29.3 (CH_2_), 29.2 (CH_2_), 28.7 (x2, Me dmphen), 26.8 (CH_2_), 22.7 (CH_2_), 20.7 (OAc), 20.6 (x2, OAc), 20.3 (OAc), 14.1 (Me), and -6.6 (PtMe, *J*_Pt_ = 466 Hz). Calculated for C_46_H_60_F_3_N_7_O_12_PtS: C, 46.54; H, 5.09; and N, 8.26. Found: C, 46.39; H, 5.14; and N, 8.20. **1-Oct,Me** (light brown solid—yield 62%). ^1^H NMR (400 MHz, 298 K, CDCl_3_): *δ* 8.49 (d, 1H, ^3^*J*_H4–H3 or H7–H8_ = 6.9, H4 or H7 dmphen), 8.49 (s, 1H, H5 triazole), 8.47 (d, 1H, ^3^*J*_H7–H8 or H4–H3_ = 6.8, H7 or H4 dmphen), 7.96–7.86 (m, 4H, H3, H5, H6, H8 dmphen), 7.06 (s, 1H, H5 imidazole), 5.85 (d, 1H, ^3^*J*_H1–H2_ = 9.3 Hz, H1 glu), 5.75 (t, 1H, ^3^*J*_H2–H3_ = 9.4 Hz, H2 glu), 5.35 (t, 1H, ^3^*J*_H3–H4_ = 9.3 Hz, H3 glu), 5.24 (t, 1H, ^3^*J*_H4–H5_ = 9.7 Hz, H4 glu), 4.25 (dd, 1H, ^2^*J*_H6–H6′_ = 12.6 Hz, ^3^*J*_H5–H6_ = 4.7 Hz, H6 glu), 4.13 (dd, 1H, ^3^*J*_H5–H6′_ = 1.8 Hz, H6′ glu), 4.06–4.00 (m, 1H, H5 glu), 3.52 (s, 3H, N-Me), 3.46 (s, 6H, Me dmphen), 3.29 (s, 3H, N-Me), 2.37 (s, 4 H, *J*_Pt_ = 75.5 Hz, ethene), 2.03 (s, 6H, OAc), 1.99 (s, 3H, OAc), 1.78 (s, 3H, OAc), 1.13 (t, 2H, ^3^*J* = 7 Hz, *J*_Pt_ = 62.2 Hz, Pt-CH_2_), 1.10–0.92 (m, 8H, CH_2_), 0.90–0.80 (m, 2H, CH_2_), 0.77 (t, 3H, Me), and 0.56 (br, 2H, CH_2_). ^13^C NMR (100 MHz, 298 K, CDCl_3_): *δ* 173.8 (C carbene), 170.8 (CO), 170.1 (CO), 169.4 (CO), 168.8 (CO), 161.0 and 160.9 (C2 and C9 dmphen), 145.8 and 145.7 (C10a and C10b dmphen), 138.8 and 138.7 (C4 and C7 dmphen), 135.9 (C5 imidazole), 128.8 (x2, C4a and C6a dmphen), 126.8 and 126.7 (C5 and C6 dmphen), 126.1 and 125.9 (C3 and C8 dmphen), 125.7 (C4 triazole), 124.4 and 122.0 (C5 triazole and C4 imidazole), 121.0 (q, CF_3_, *J*_C–F_ = 317 Hz), 85.1 (C1 glu), 74.8, 73.3, 70.0 and 67.6 (C2–C5 glu), 61.5 (C6 glu), 37.1 (nMe), 36.4 (nMe), 33.3 (CH_2_), 31.8 (CH_2_), 30.9 and 30.8 (ethene), 30.0, 29.2, 29.1 and 28.9 (x2) (3 CH_2_ and Me dmphen), 22.6 (CH_2_), 20.7 (OAc), 20.6 (x2, OAc), 20.3 (OAc), 14.1 (Me), and 13.8 (PtCH_2_, *J*_Pt_ = 458 Hz). Calculated for C_46_H_60_F_3_N_7_O_12_PtS: C, 46.54; H, 5.09; and N, 8.26. Found: C, 46.73; H, 4.99; and N, 8.35. **1-Oct,Oct** (light brown solid—yield 71%). ^1^H NMR (400 MHz, 298 K, CDCl_3_): *δ* 8.61 (s, 1H, H5 triazole), 8.49 (d, 1H, ^3^*J*_H4–H3 or H7–H8_ = 8.7 Hz, H4 or H7 dmphen), 8.47 (d, ^3^*J* _H7–H8 or H4–H3_ = 8.9 Hz 1H, H7 or H4 dmphen), 7.98–7.82 (m, 4H, H3, H5, H6, H8 dmphen), 7.06 (s, 1H, H5 imidazole), 5.89 (d, 1H, ^3^*J*_H1–H2_ = 9.4 Hz, H1 glu), 5.82 (t, 1H, ^3^*J*_H2–H3_ = 9.2 Hz, H2 glu), 5.35 (t, 1H, ^3^*J*_H3–H4_ = 9.2 Hz, H3 glu), 5.25 (t, 1H, ^3^*J*_H4–H5_ = 9.7 Hz, H4 glu), 4.26 (dd, 1H, ^2^*J*_H6–H6′_ = 12.7 Hz, ^3^*J*_H5–H6_ = 4.6 Hz, H6 glu), 4.14 (dd, 1H, ^3^*J*_H5–H6′_ = 2.1 Hz, H6′ glu), 4.09–3.99 (m, 1H, H5 glu), 3.65 (br, 2H, N-CH_2_), 3.53 (s, 3H, N-Me), 3.46 (s, 6H, Me dmphen), 2.36 (s, 4 H, *J*_Pt_ = 73 Hz, ethene), 2.04 (s, 3H, OAc), 2.03 (s, 3H, OAc), 1.99 (s, 3H, OAc), 1.78 (s, 3H, OAc), 1.40–1.20 (m, 10H, CH_2_), 1.13 (t, 2H, Pt-CH_2_), 1.10–0.90 (m, 10H, CH_2_), 0.91 (t, 3H, ^3^*J* = 7 Hz, Me), 0.77 (t, 3H, Me), and 0.55 (br, 2H, CH_2_). ^13^C NMR (125 MHz, 298 K, CDCl_3_): *δ* 173.6 (C carbene, *J*_Pt_ = 702 Hz), 170.8 (CO), 170.1 (CO), 169.4 (CO), 168.8 (CO), 160.8 (x2, C2 and C9 dmphen), 145.8 (x2, C10a and C10b dmphen), 138.7 and 138.6 (C4 and C7 dmphen), 135.8 (C4 imidazole), 128.8 and 128.7 (C4a and C6a dmphen), 126.8 and 126.6 (C5 and C6 dmphen), 126.6 and 126.3 (C3 and C8 dmphen), 126.1 (C4 triazole), 124.8 (C5 triazole), 118.9 (C5 imidazole), 85.0 (C1 glu), 74.7, 73.5, 69.9 and 67.7 (C2–C5 glu), 61.5 (C6 glu), 49.4 (NCH_2_), 36.4 (nMe), 33.3 (CH_2_), 31.8 (x2, CH_2_), 30.9 (x2, ethene), 30.6, 29.9, 29.4, 29.3, 29.2, 29.1 and 28.9 (x2) (6 CH_2_ and Me dmphen), 26.8 (CH_2_), 22.7 (x2, CH_2_), 20.8 (OAc), 20.7 (x2, OAc), 20.4 (OAc), 14.2 (Me), 14.1 (Me), and 13.7 (PtCH_2_). Calculated for C_53_H_74_F_3_N_7_O_12_PtS: C, 49.53; H, 5.80; and N, 7.63. Found: C, 49.32; H, 5.71; and N, 7.52.

### 3.5. In-Solution Stability of **1-R,R’** Complexes

The stability of the **1-R,R’** complexes in DMSO was studied by dissolving 5 mg of each complex in 250 μL of DMSO-d_6_ and then diluting 200 μL of this solution with 300 μL of DMSO-d_6_. The stability of the **1-R,R’** complexes in aqueous solvents was studied by dissolving 5 mg of each complex in 250 μL of DMSO-d_6_ and then diluting 50 μL of this solution with 500 μL of D_2_O. The ^1^H-NMR spectra of the resulting solutions were recorded over time.

UV–vis absorption spectra of **1-Me,Me** were collected at 25 °C on a JASCO V-750 UV–vis spectrophotometer in the range of 240–500 nm, using a platinum compound concentration of 50 μM in 100% DMSO as well as under the crystallization conditions of 20% ethylene glycol, 0.1 M sodium acetate buffer at pH 4.0, and 0.6 M sodium nitrate and 2.0 M sodium formate and 0.1 M Hepes at pH 7.5, in the absence and in the presence of HEWL. The HEWL:**1-Me,Me** molar ratio was 1:3. Each measurement was repeated three times.

### 3.6. Partition Coefficients

Partition coefficients for the platinum complexes were determined in triplicate in an *n*-octanol/water system, with different ratios (1:1, 1:2, 2:1). Each complex was dissolved in *n*-octanol at the concentrations 16.7, 12.5, or 10 μM and subsequently, an appropriate volume of water was added. The mixtures were shaken mechanically for 1 h to ensure the distribution between the two solvent phases. The samples were then centrifuged (13,000 rpm, 10 min). Afterwards, the platinum concentration was determined in the octanol phases by UV–vis spectrophotometry, after collecting a calibration line for each complex. Results are expressed as the logarithm of the partition coefficient of octanol/water (log P_o/w_), which is the logarithm of the concentration of platinum in the *n*-octanol divided by its concentration in the aqueous layer.

### 3.7. DNA Binding Assays

The ethidium bromide (EB) displacement assay was performed on a HORIBA FluoroMax-4 spectrofluorometer equipped with a thermostat bath. Calf thymus DNA was incubated with EB in 0.05 M of ammonium acetate at pH 7.5, at a DNA:EB molar ratio of 1:5 for 30 min in the dark at room temperature. Then, the fluorescence quenching of this complex was evaluated by adding to it increasing amounts of Pt compounds dissolved in DMSO (20 mM). Samples were equilibrated for 5 min before collecting each spectrum. The other experimental settings comprised the following: a 1.0 cm quartz cell, 5.0 nm excitation/emission slit, 560–750 nm range, and 50 nm minutes^−1^ scanning speed. Data were obtained as the average of three independent measurements.

### 3.8. Crystallization of the Adducts Formed by the Reaction of **1-Me,Me** with HEWL

HEWL crystals were grown using the hanging drop vapour diffusion method and the following reservoirs:

(a) 20% ethylene glycol, 0.10 M sodium acetate at pH 4.0, and 0.60 M sodium nitrate
(b) 2.0 M sodium formate and 0.1 M HEPES at pH 7.5.

The crystals of the protein adduct with **1-Me,Me** were obtained by the soaking procedure. The HEWL crystals were soaked in a solution consisting of 83% reservoir, 17% DMSO, and 3.3 mM **1-Me,Me**.

### 3.9. Structure Solution and Refinement of Structures **A** and **B**

The structures of the Pt–HEWL adducts were solved by the molecular replacement method, using the HEWL coordinates deposited in the PDB under the accession code 193L [40] as a model. Refinements were carried out with a CCP4 REFMAC5 [46], and the model building, adjustments, and inspection of the electron density maps was manually carried out using WinCoot [47].

The Pt binding sites were unambiguously identified by comparing 2Fo–Fc, residual Fo–Fc, and anomalous difference electron density maps. The two structures were refined to the *R*-factor/*R*_free_ values of 0.187/0.219 and 0.184/0.218, respectively. The details of the crystallographic and refinement parameters are given in Table S2. The refined models and structure factors were deposited in the Protein Data Bank under the accession codes 8BOY and 8BOV. The coordinates and structure factors, including anomalous data, were provided to the reviewers and editor for the review process.

### 3.10. Cell Culture and Cytotoxicity

The immortalized human keratinocytes (HaCaT) were from Innoprot. Human The A431 epidermoid carcinoma, murine BALB/c-3T3, and SVT2 fibroblasts were from ATCC. Cells were cultured in Dulbecco’s modified Eagle’s medium (DMEM) (Sigma-Aldrich, St. Louis, MO, USA), supplemented with 10% foetal bovine serum (HyClone), 2 mM L-glutamine, and antibiotics, all from Sigma-Aldrich, under a 5% CO_2_-humidified atmosphere at 37 °C. To test the effects of the complexes on cell viability, cells were seeded at a density of 2.5 × 10^3^ cells per well in 96-well plates. After 24 h, cells were incubated with increasing concentrations (from 0.1 to 50 μM) of the four complexes. After 48 h, cell viability was assessed by the MTT assay, as previously reported in [43]. Cell viability was expressed as the percentage of viable cells in the presence of the Pt complexes compared to the controls, represented by untreated cells and cells supplemented with identical volumes of DMSO. Each sample was tested in three independent analyses, each carried out in triplicate.

### 3.11. Uptake Experiments

To study the uptake of the complexes, SVT2 and A431 were incubated for 48 h in the presence of **1-Me,Me** and **1-Oct,Me**, respectively, at their IC_50_ concentrations. At the end of incubation, Pt determination was performed by inductively coupled plasma mass spectrometry (ICP-MS, Aurora M90; Bruker, Ettlingen/Leipzig, Germany) in the “Normal Sensitivity” mode. Calibration curves for the quantification of Pt ranged from 0.1 to 100 μg/L and were constructed daily by the analysis of the standard solutions prepared immediately before analysis. All standards used for analysis were prepared in HNO_3_ solution (2%, *v*/*v*). The internal standards were ^89^Y and ^115^In in both the calibration curve and sample analyses. The linearity was acceptable with an R^2^ value greater than 0.9996. The treatment of the cells involved a wet digestion: after centrifugation, cell samples were wet digested with 1 mL of ultrapure HNO_3_ (67–69%, *v*/*v*). The mixture was gently boiled over a water bath (90 °C) for 3 h until a clear solution was obtained. After cooling, HNO_3_ solution (2%, *v*/*v*) was added up to a final volume of 10 mL. The obtained solutions were analysed by ICP-MS. Under this condition, the quantification limit for Pt was equal to 0.001 ng Pt/10^6^ cells.

### 3.12. Western Blot Analysis

The SVT2 were seeded on six-well plates at a density of 2.5 × 10^5^ cells/well, whereas A431 was seeded at a density of 3 × 10^5^ cells/well. After 24 h, cells were treated with 9.8 μM of **1-Me,Me** or 2.3 μM **1-Oct,Me**. After 48 h of incubation, Western blot analyses were performed by using pro-caspase 9 (Cell Signaling Technology, Danvers, MA, USA) and pro-caspase 3 (Abcam, Cambridge, MA, USA) antibodies, as reported by Del Giudice et al. [48] Protein intensity levels were normalized using β-actin (Sigma-Aldrich, St. Louis, MO, USA). The chemiluminescence detection system was purchased from Bio-Rad (Hercules, CA, USA).

### 3.13. Statistical Analysis

All experiments were performed in triplicate. The results are presented as the mean of the results obtained after three independent experiments and compared by one-way ANOVA according to Bonferroni’s method (post hoc), obtained using GraphPad Prism for Windows, version 6.01.

## 4. Conclusions

This study demonstrates the versatility of platinum(II) anticancer agents with a coordination number of five. Previous studies on methyl/carbene complexes with trigonal bipyramidal geometry [14] have demonstrated that their structural diversity is enriched by introducing variable-length alkyl groups in strategic positions of the coordination environment. This allows for a comparison of biological activity among complexes with different lipophilicity, revealing different behaviours of the drug being tested, depending on the cell system being analysed. The tested drug was internalized in cancer cells and able to activate the apoptotic pathway. One might speculate that the activity of the complexes may reside in the different compositions of their plasma membranes. Accordingly, changes in the structural arrangements of membrane lipids may influence their lipid core and consequently their overall surface properties [49,50]. The structural study also revealed a variety of interactions between the metal fragment and the model protein HEWL. In one of these, the metal complex retained its 5C geometry and established non-covalent interactions with the protein. These data, along with the observation that the same complex is stable for days in an aqueous solvent, demonstrate the great structural robustness of this class of compounds and encourage further study towards understanding and optimization.

## Figures and Tables

**Table 1:** Octanol/water partition coefficients.

Complex	Log P_o/w_
**1-Me,Me**	0.42
**1-Me,Oct**	0.74
**1-Oct,Me**	1.14
**1-Oct,Oct**	0.80

**Table 2:** IC_50_ values (μM) obtained for Pt complexes on HaCaT, A431, BALB/c-3T3 and SVT2 cells after 48 h incubation. The selectivity indexes (SI) are indicated by the ratios between the IC_50_ values of the immortalized cells and cancer cells.

	HaCaT	A431	SI	BALB-c/3T3	SVT2	SI
**1-Me,Me**	8.4 ± 1.7	20 ± 2	0.42	26 ± 1	9.8 ± 0.3	2.65
**1-Me,Oct**	8.0 ± 0.1	7.3 ± 0.4	1.1	8.3 ± 0.4	7.6 ± 1.1	1.09
**1-Oct,Me**	6.4 ± 1.0	2.3 ± 0.1	2.78	7.1 ± 0.1	8.6 ± 0.6	0.83
**1-Oct,Oct**	6.4 ± 0.8	5.0 ± 0.6	1.28	7.3 ± 0.7	4.5 ± 1.3	1.62

**Table 3:** The uptake of **1-Me,Me** and **1-Oct,Me** in cancer cells. Cells were incubated with either **1-Me,Me** or **1-Oct,Me**, with each one tested at its IC_50_ value (9.8 μM **1-Me,Me** on SVT2 and 2.3 μM **1-Oct,Me** on A431). After 48 h, cells were analysed by ICP analysis, as described in the Materials and Methods section. Data shown are the means ± S.E. of three independent experiments.

Samples	Pt ng/10^6^ cells
Untreated SVT2	<0.0001
SVT2 incubated with **1-Me,Me**	0.772 ± 0.010
Untreated A431	<0.0001
A431 incubated with **1-Oct,Me**	14.3 ± 1.3

## Data Availability

Data is contained within the article or supplementary material.

## Supplementary Materials

The following supporting information can be downloaded at: https://www.mdpi.com/article/10.3390/ijms24032369/s1.
