# Supplementary material for: Impact of Hydrophobic Chains in Five-Coordinate Glucoconjugate Pt(II) Anticancer Agents
Source: Int J Mol Sci. 2023 Jan 25;24(3):2369. doi: 10.3390/ijms24032369 (PMC9916762; doi:10.3390/ijms24032369)
Supplement: Supplementary file 1 [file ijms-24-02369-s001.zip › ijms-2072264-supplementary.pdf]

*Supplementary Materials*

# Impact of hydrophobic chains in five-coordinate glucoconjugate Pt(II) anticancer agents

**Alfonso Annunziata [1], Maria Elena Cucciolito [2,3], Giarita Ferraro [2], Paola Imbimbo [2], Vincenzo Langellotti [2], Alessandra Marano,[2] Massimo Melchiorre [2,3], Gabriella Tito [2], Marco Trifuoggi,[2] Daria Maria Monti [2], Antonello Merlino [2] and Francesco Ruffo [2,3,*]**

1 Institute Parisien de Chimie Moléculaire, Sorbonne Université, Campus Pierre et Marie Curie - 4 place Jussieu, 75005, Paris, France
2 Dipartimento di Scienze Chimiche, Università degli Studi di Napoli Federico II, Complesso Universitario di Monte S. Angelo, Via Cintia 21, 80126, Napoli, Italy
3 Consorzio Interuniversitario di Reattività Chimica e Catalisi (CIRCC), Via Celso Ulpiani 27, 70126, Bari, Italy
* Correspondence: ruffo@unina.it

## Table of Content

**Table S1.** - IUPAC names of the new compounds……2
**Figure S1** - $^1$H NMR spectrum of **Im-Oct** …… 3
**Figure S2** - $^{13}$C NMR spectrum of **Im-Oct** …… 3
**Figure S3** - $^1$H NMR spectrum of **2-Oct** …… 4
**Figure S4** - $^{13}$C NMR spectrum of **2-Oct** …… 4
**Figure S5** - $^1$H NMR spectrum of **1-Me,Me** …… 5
**Figure S6** - $^{13}$C NMR spectrum of **1-Me,Me** …… 5
**Figure S7** - $^1$H NMR spectrum of **1-Me,Oct** …… 6
**Figure S8** - $^{13}$C NMR spectrum of **1-Me,Oct** …… 6
**Figure S9** - $^1$H NMR spectrum of **1-Oct,Me** …… 7
**Figure S10** - $^{13}$C NMR spectrum of **1-Oct,Me** ……7
**Figure S11** - $^1$H NMR spectrum of **1-Oct,Oct** …… 8
**Figure S12** - $^{13}$C NMR spectrum of **1-Oct,Oct** …… 8
**Figure S13** - $^1$H NMR spectra of **1-Me,Me** in DMSO-$d_6$ over time …… 9
**Figure S14** - $^1$H NMR spectra of **1-Me,Oct** in DMSO-$d_6$ over time …… 10
**Figure S15** - $^1$H NMR spectra of **1-Oct,Me** in DMSO-$d_6$ over time …… 11
**Figure S16** - $^1$H NMR spectra of **1-Oct,Oct** in DMSO-$d_6$ over time …… 12
**Figure S17** - $^1$H NMR spectra of **1-Me,Me** in $D_2O$:DMSO-$d_6$ 10:1 over time …… 13
**Figure S18** - UV–vis spectra **1-Me,Me** over time in different conditions …… 14
**Table S2** - Data collection and refinement statistics of crystal structures …… 15
**Figure S19** - Effect of complexes on cell viability …… 16

**Table S1. -** IUPAC names of the new compounds

| Label | IUPAC name |
|---|---|
| **Im-Oct** | 1*H*-Imidazolium, 1-methyl-3-octyl-4-[[1-[2,3,4,6-tetrakis-*O*-(acetyl)-β-D-glucopyranosyl]-1*H*-1,2,3-triazol-4-yl], iodide |
| **2-Oct** | Platinum, iodo(2,9-dimethyl-1,10-phenanthroline-$N^1$,$N^{10}$)($\eta^2$-ethene)octyl |
| **1-Me-Me** | Platinum(1+), (2,9-dimethyl-1,10-phenanthroline- $N^1$,$N^{10}$)($\eta^2$-ethene)methyl[1,3-dihydro-1,3-dimethyl-4-[1-[2,3,4,6-tetrakis-*O*-(acetyl)-β-D-glucopyranosyl]-1*H*-1,2,3-triazol-4-yl]-2*H*-imidazol-2-ylidene], 1,1,1-trifluoromethanesulfonate |
| **1-Me,Oct** | Platinum(1+), (2,9-dimethyl-1,10-phenanthroline- $N^1$,$N^{10}$)($\eta^2$-ethene)methyl[1,3-dihydro-1-methyl-3-octyl-4-[1-[2,3,4,6-tetrakis-*O*-(acetyl)-β-D-glucopyranosyl]-1*H*-1,2,3-triazol-4-yl]-2*H*-imidazol-2-ylidene], 1,1,1-trifluoromethanesulfonate |
| **1-Oct,Me** | Platinum(1+), (2,9-dimethyl-1,10-phenanthroline- $N^1$,$N^{10}$)($\eta^2$-ethene)octyl[1,3-dihydro-1,3-dimethyl-4-[1-[2,3,4,6-tetrakis-*O*-(acetyl)-β-D-glucopyranosyl]-1*H*-1,2,3-triazol-4-yl]-2*H*-imidazol-2-ylidene], 1,1,1-trifluoromethanesulfonate |
| **1-Oct,Oct** | Platinum(1+), (2,9-dimethyl-1,10-phenanthroline- $N^1$,$N^{10}$)($\eta^2$-ethene)octyl[1,3-dihydro-1-methyl-3-octyl-4-[1-[2,3,4,6-tetrakis-*O*-(acetyl)-β-D-glucopyranosyl]-1*H*-1,2,3-triazol-4-yl]-2*H*-imidazol-2-ylidene], 1,1,1-trifluoromethanesulfonate |

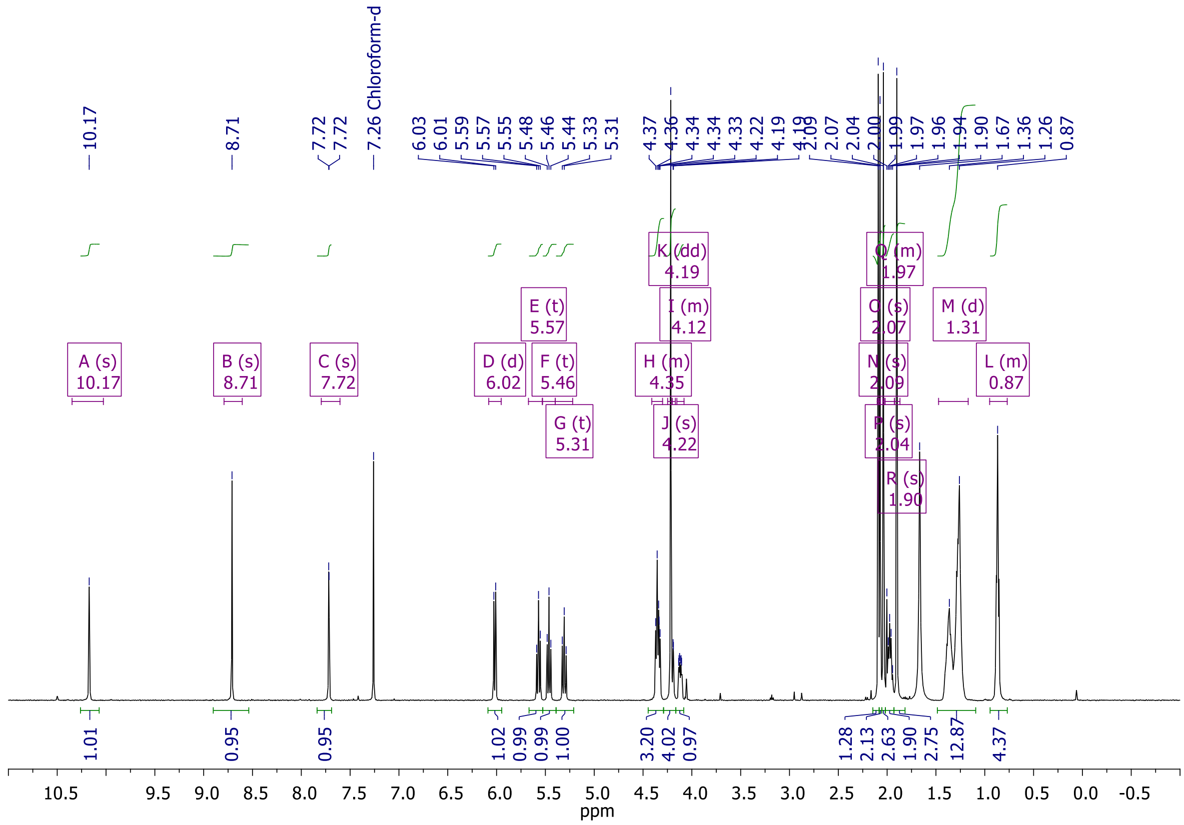


**Figure S1** - $^{1}$H NMR spectrum of **Im-Oct** (500 MHz, 298 K, $CDCl_3$).

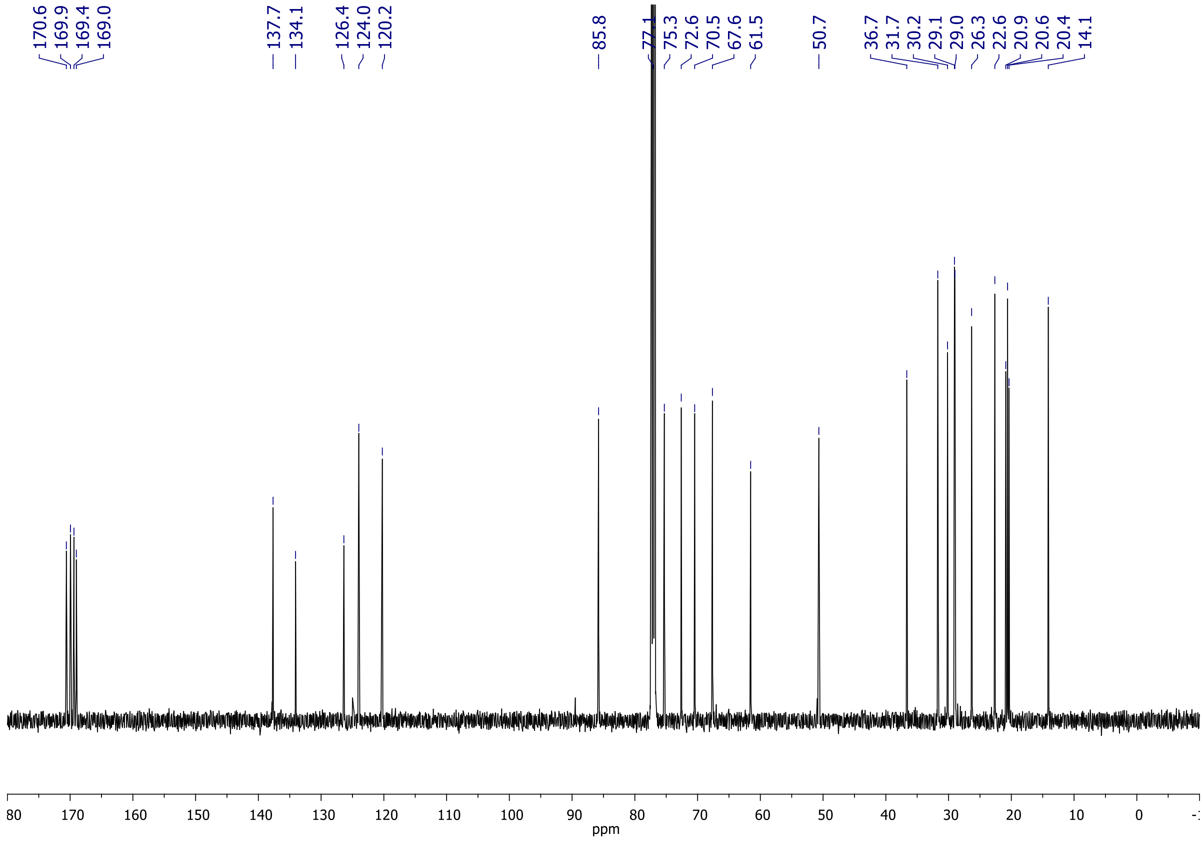


**Figure S2** - $^{13}$C NMR spectrum of **Im-Oct** (125.7 MHz, 298 K, $CDCl_3$).

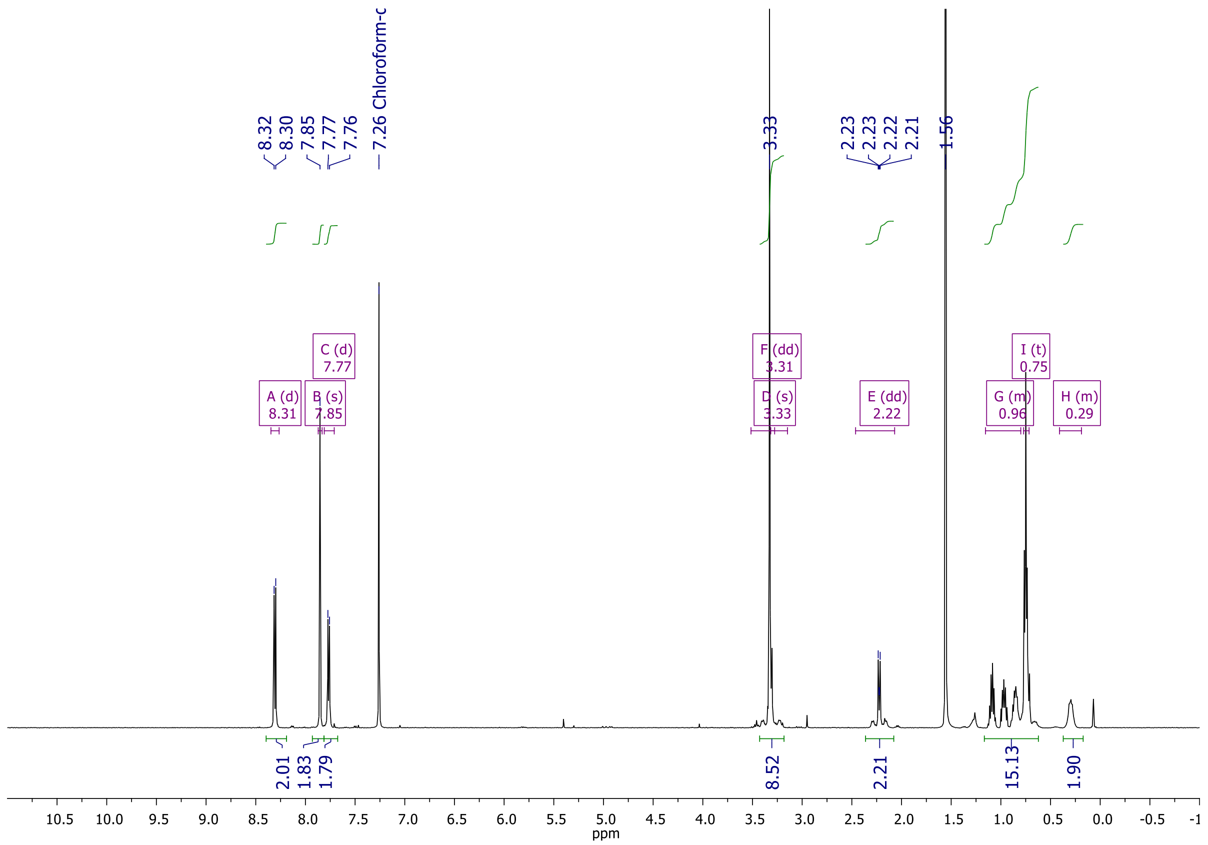


**Figure S3** - $^{1}$H NMR spectrum of **2-Oct** (500 MHz, 298 K, $CDCl_3$).

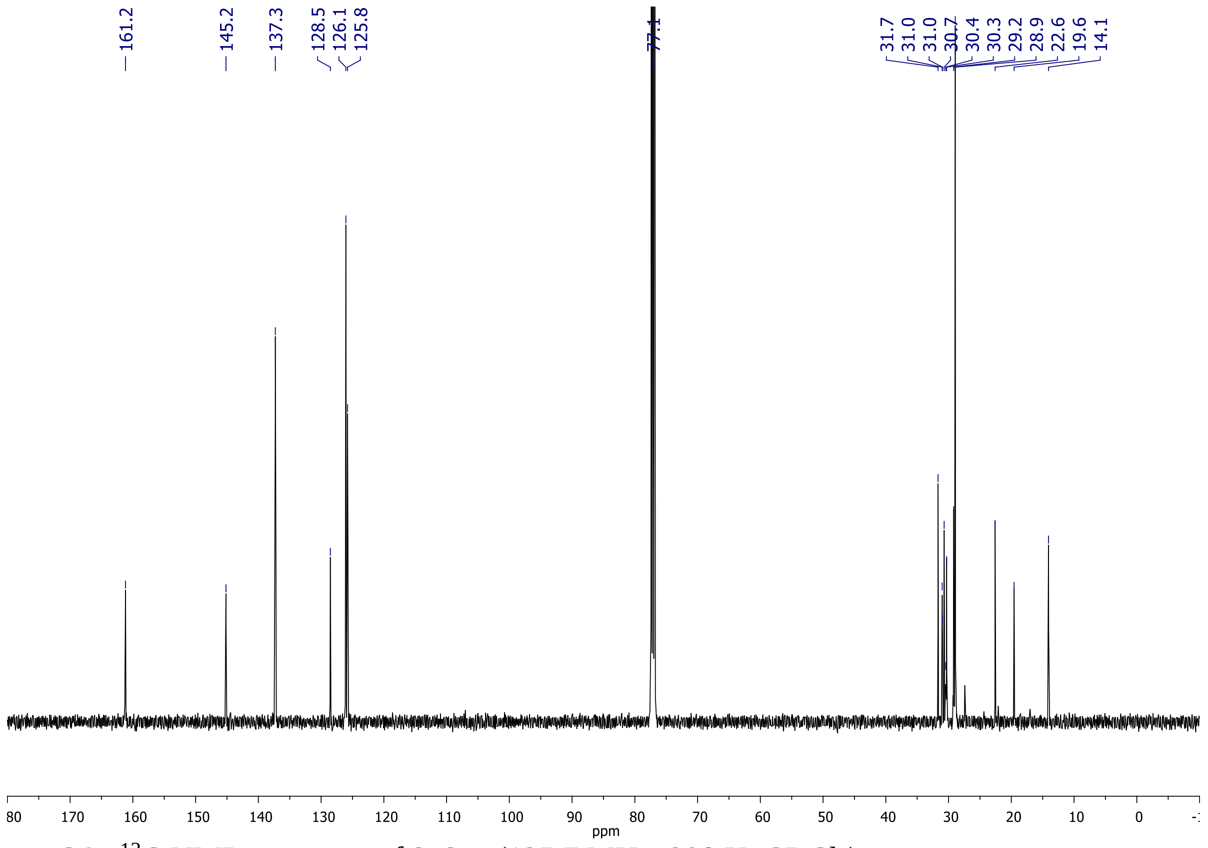


**Figure S4** - $^{13}$C NMR spectrum of **2-Oct** (125.7 MHz, 298 K, $CDCl_3$).

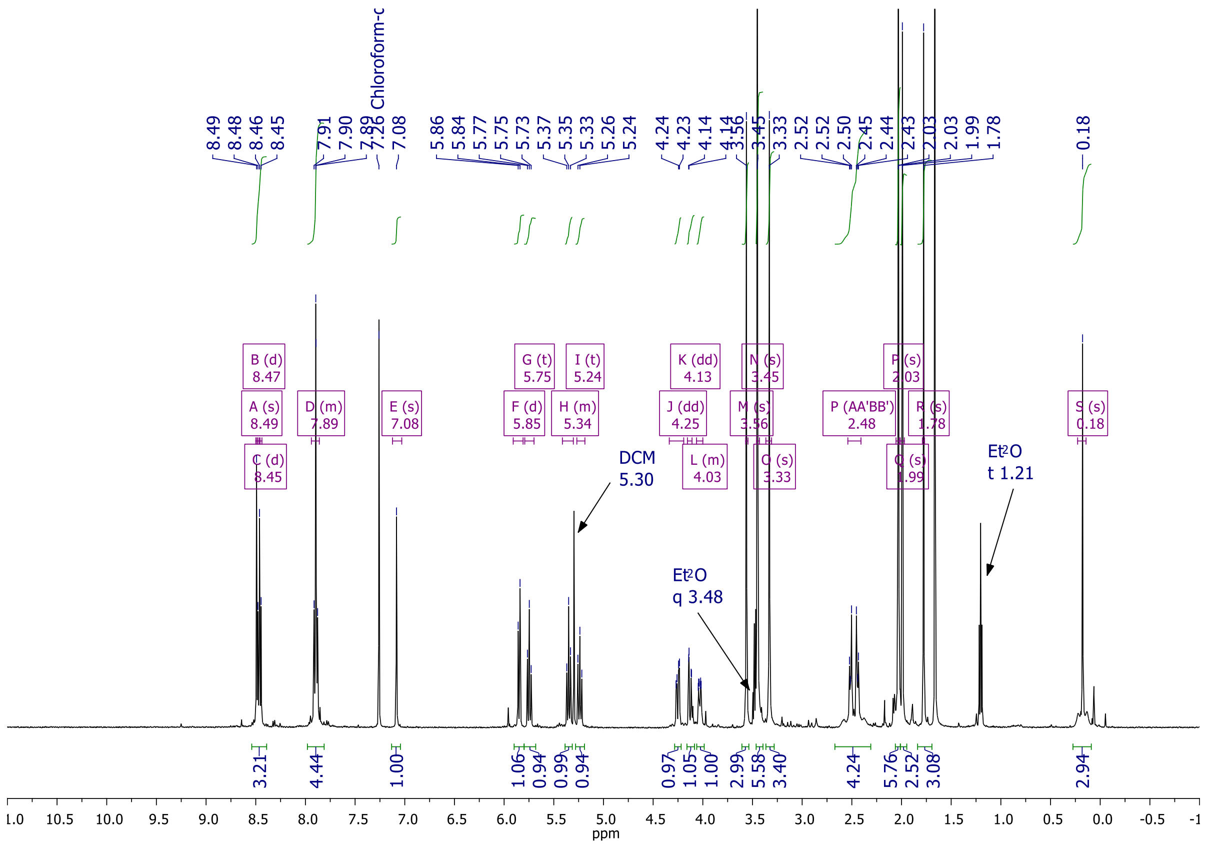


**Figure S5** - $^{1}$H NMR spectrum of **1-Me,Me** (500 MHz, 298 K, $CDCl_3$).

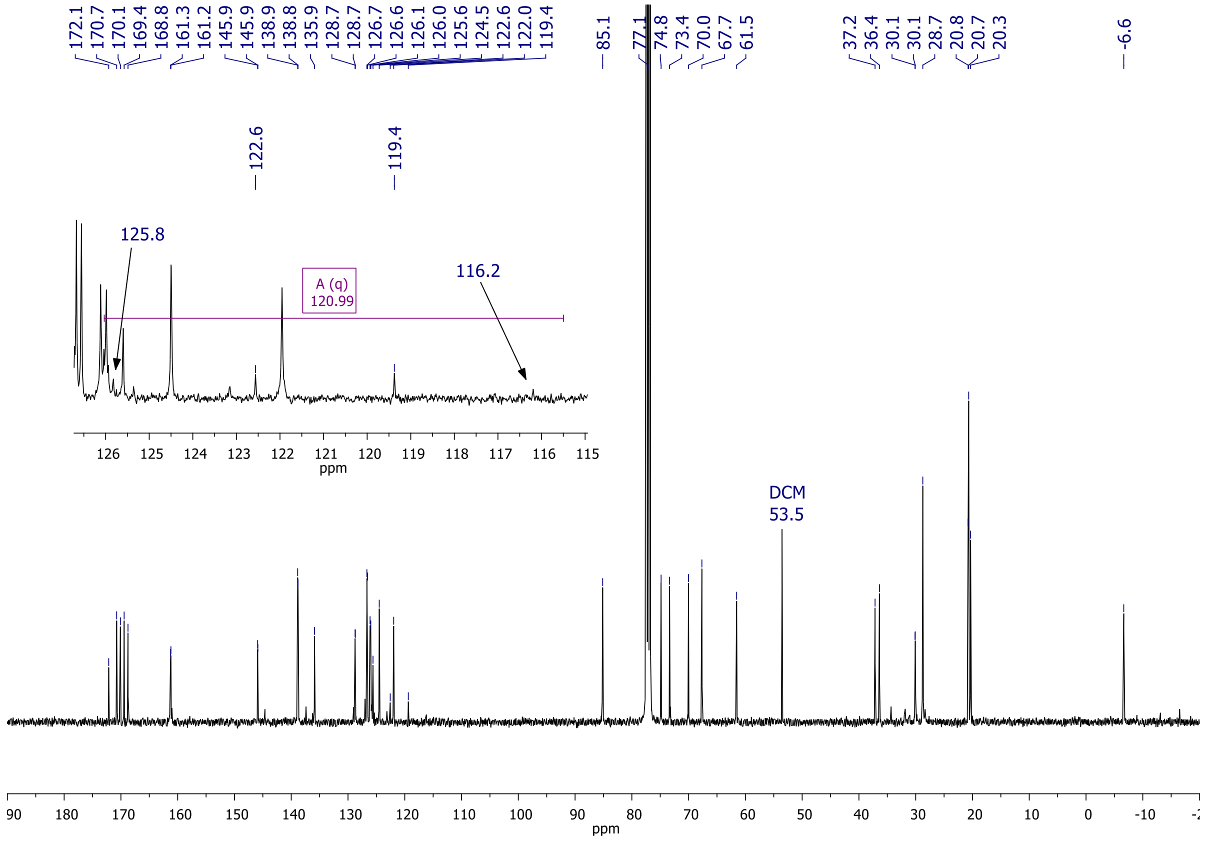


**Figure S6** - $^{13}$C NMR spectrum of **1-Me,Me** (100.6 MHz, 298 K, $CDCl_3$).

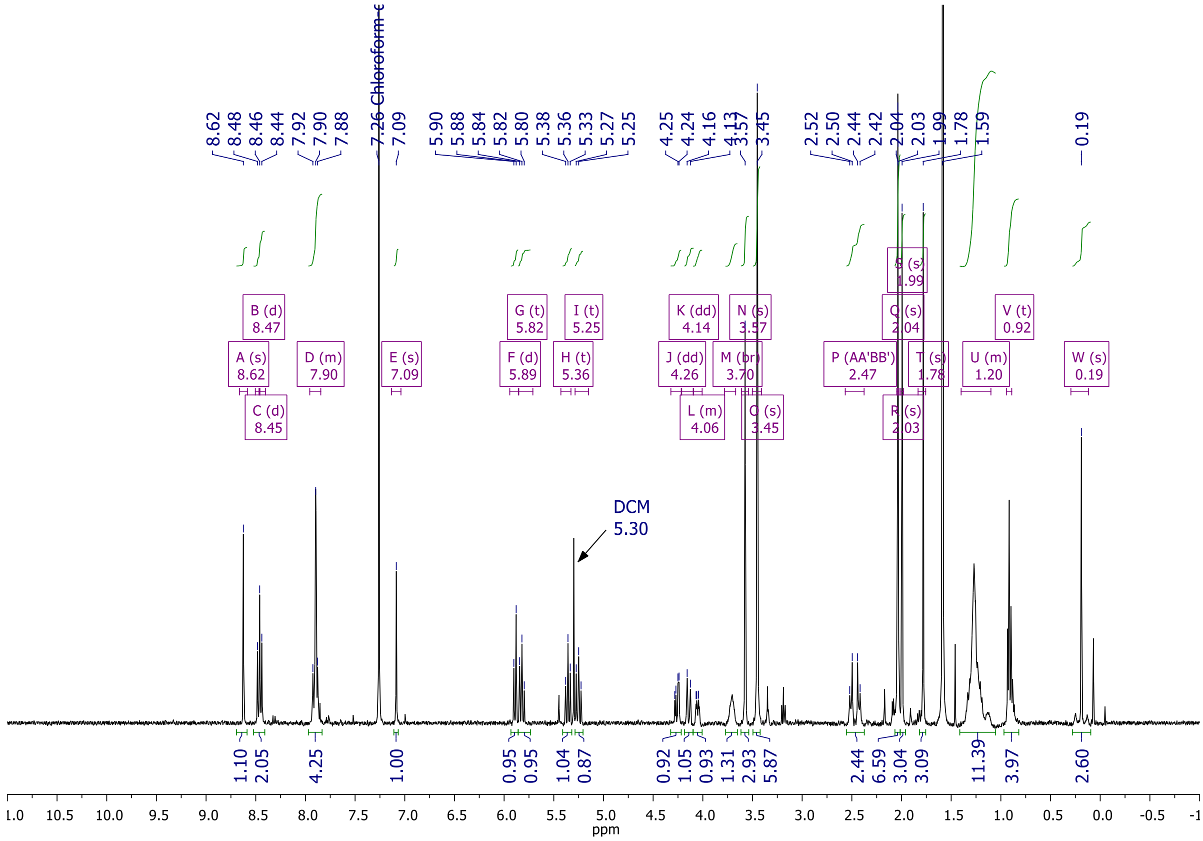


**Figure S7** - $^{1}$H NMR spectrum of **1-Me,Oct** (400 MHz, 298 K, $CDCl_3$).

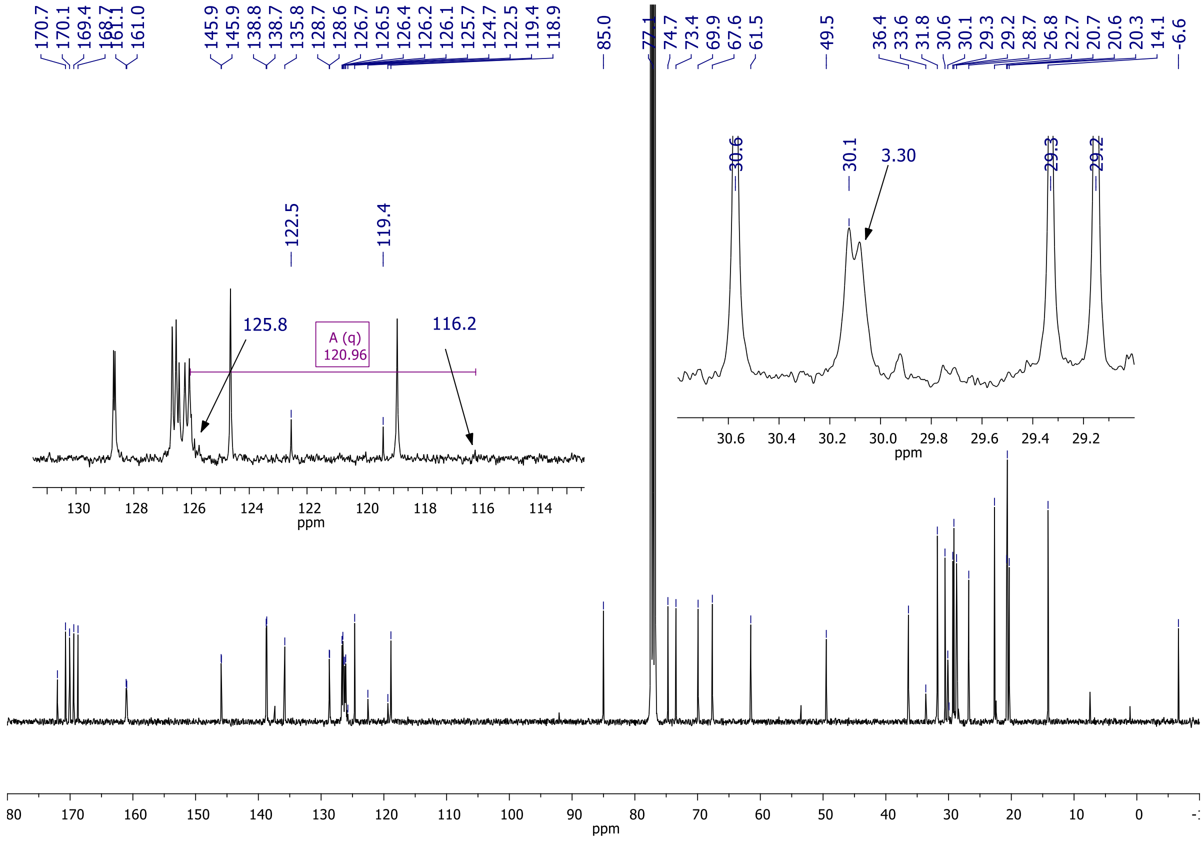


**Figure S8** - $^{13}$C NMR spectrum of **1-Me,Oct** (100.6 MHz, 298 K, $CDCl_3$).

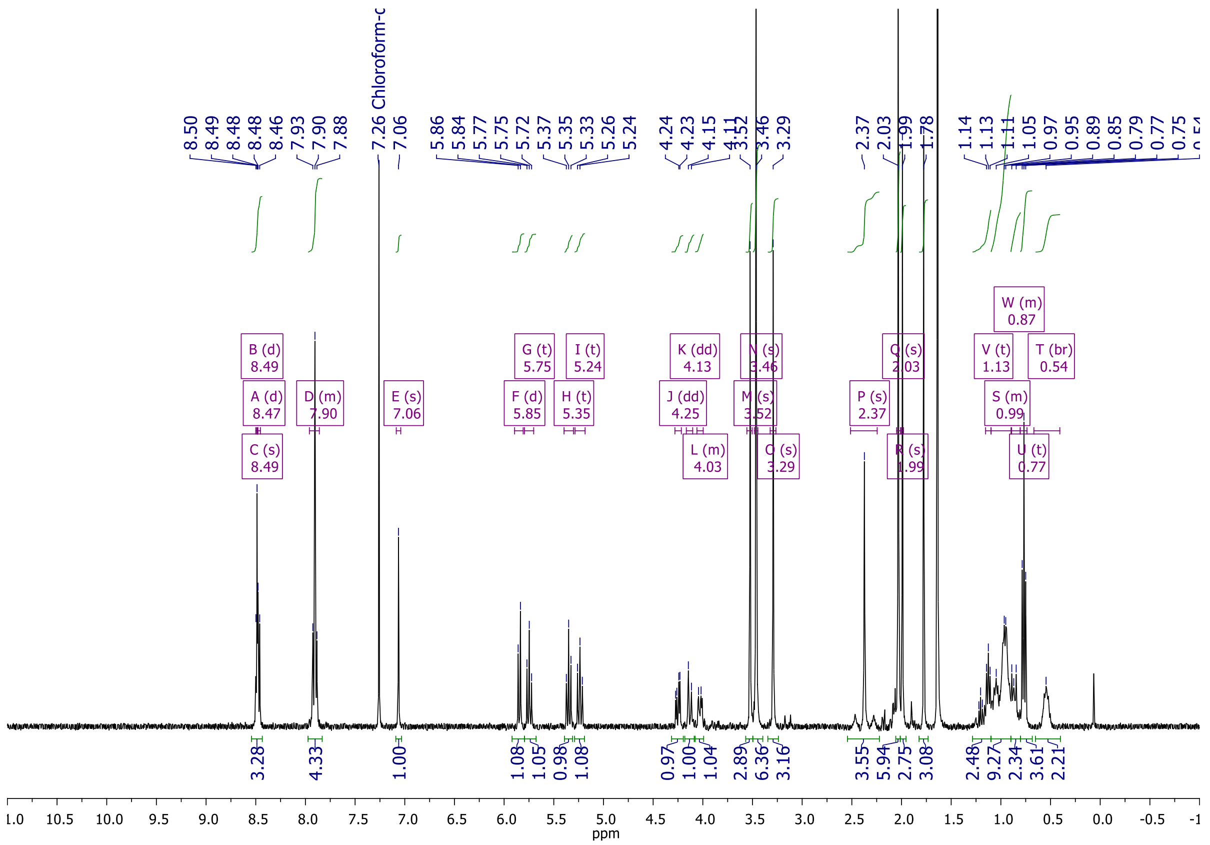


**Figure S9** - $^{1}$H NMR spectrum of **1-Oct,Me** (500 MHz, 298 K, $CDCl_3$).

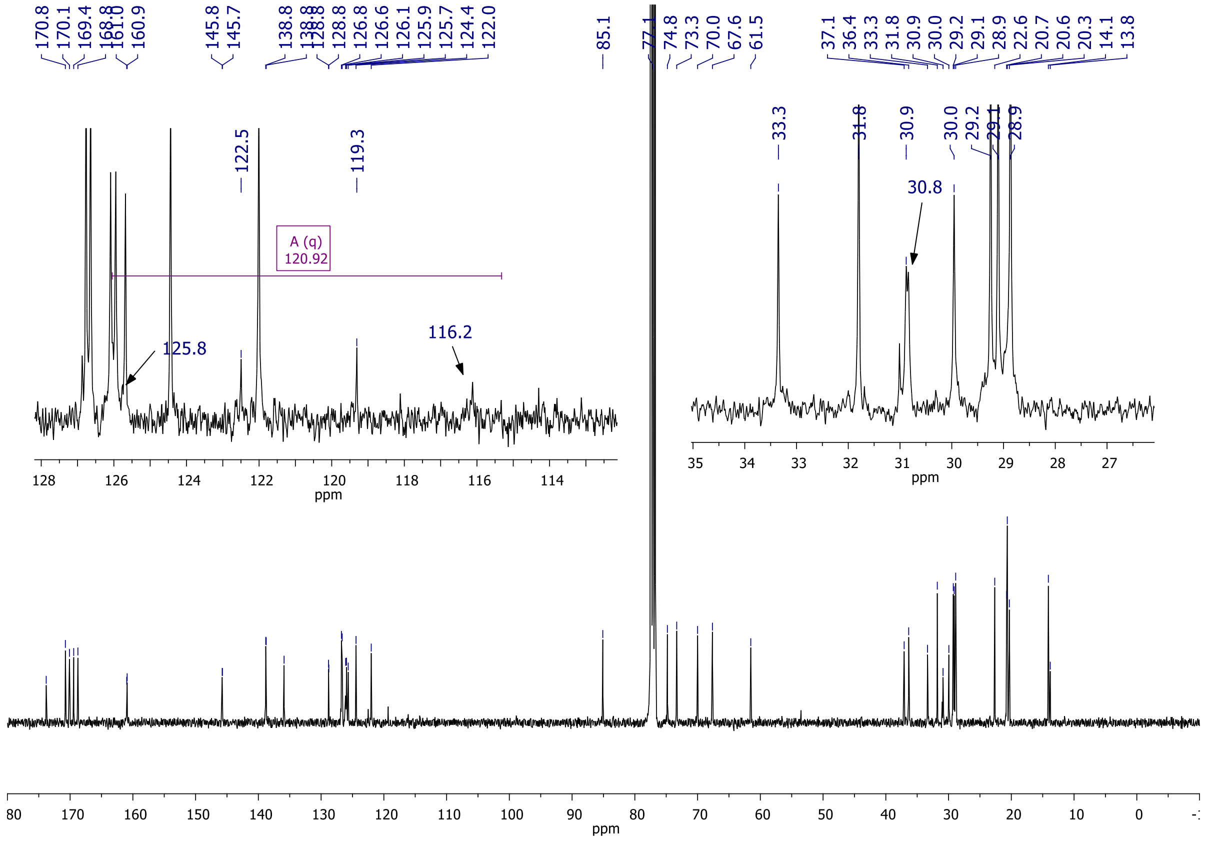


**Figure S10** - $^{13}$C NMR spectrum of **1-Oct,Me** (100.6 MHz, 298 K, $CDCl_3$).

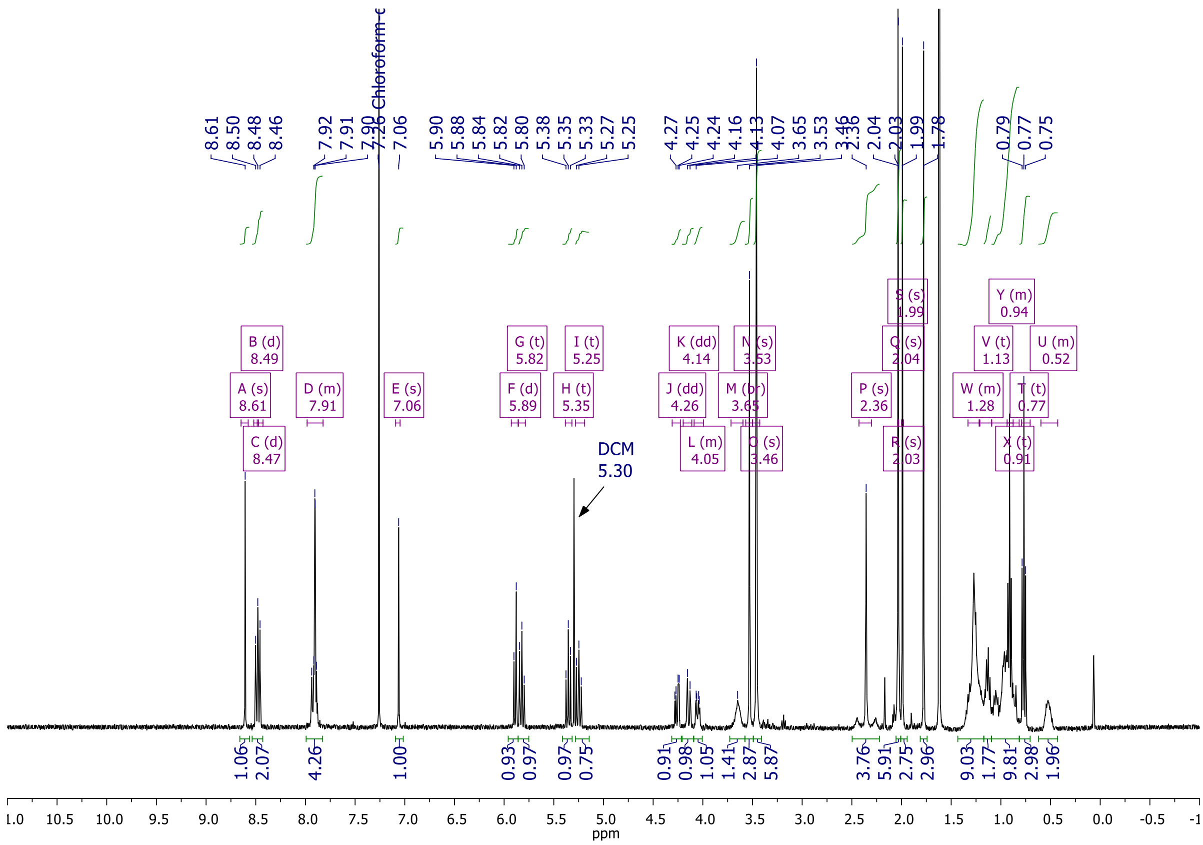


**Figure S11** - $^{1}H$ NMR spectrum of **1-Oct,Oct** (400 MHz, 298 K, $CDCl_3$).

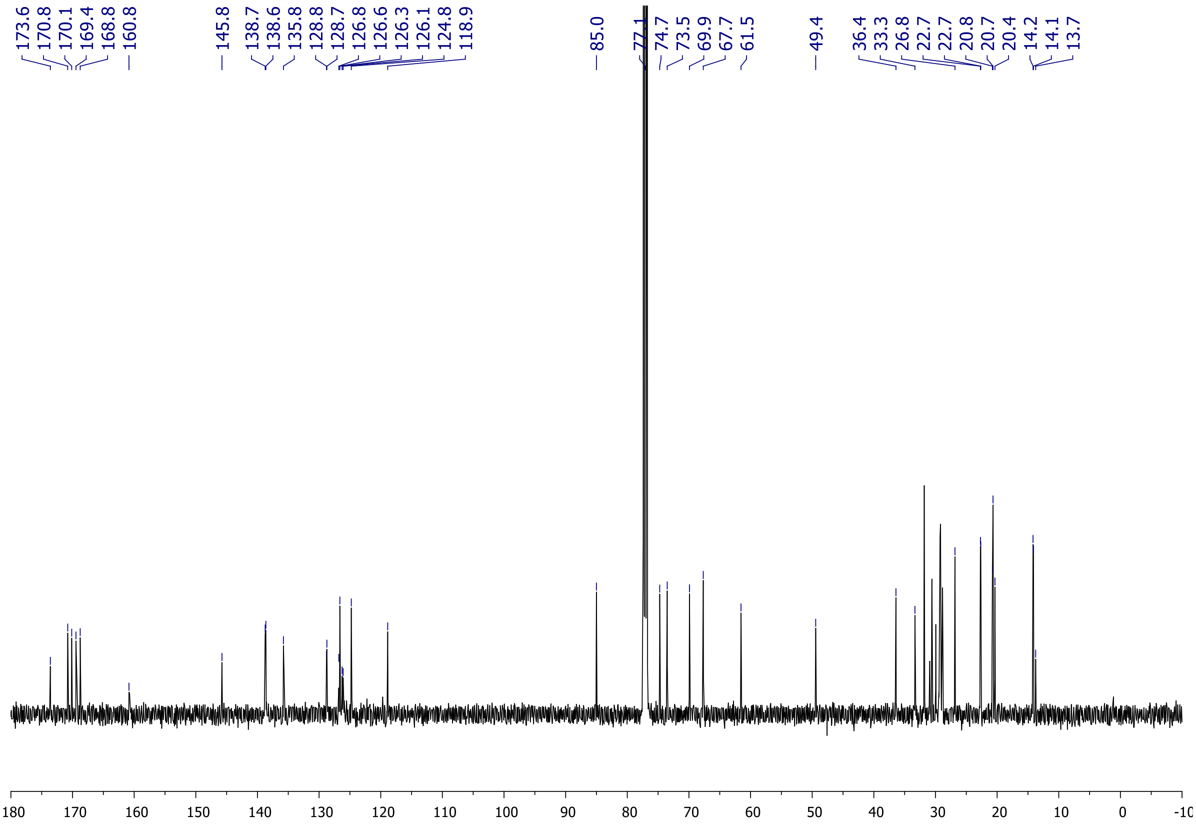


**Figure S12** - $^{13}C$ NMR spectrum of **1-Oct,Oct** (125.7 MHz, 298 K, $CDCl_3$).

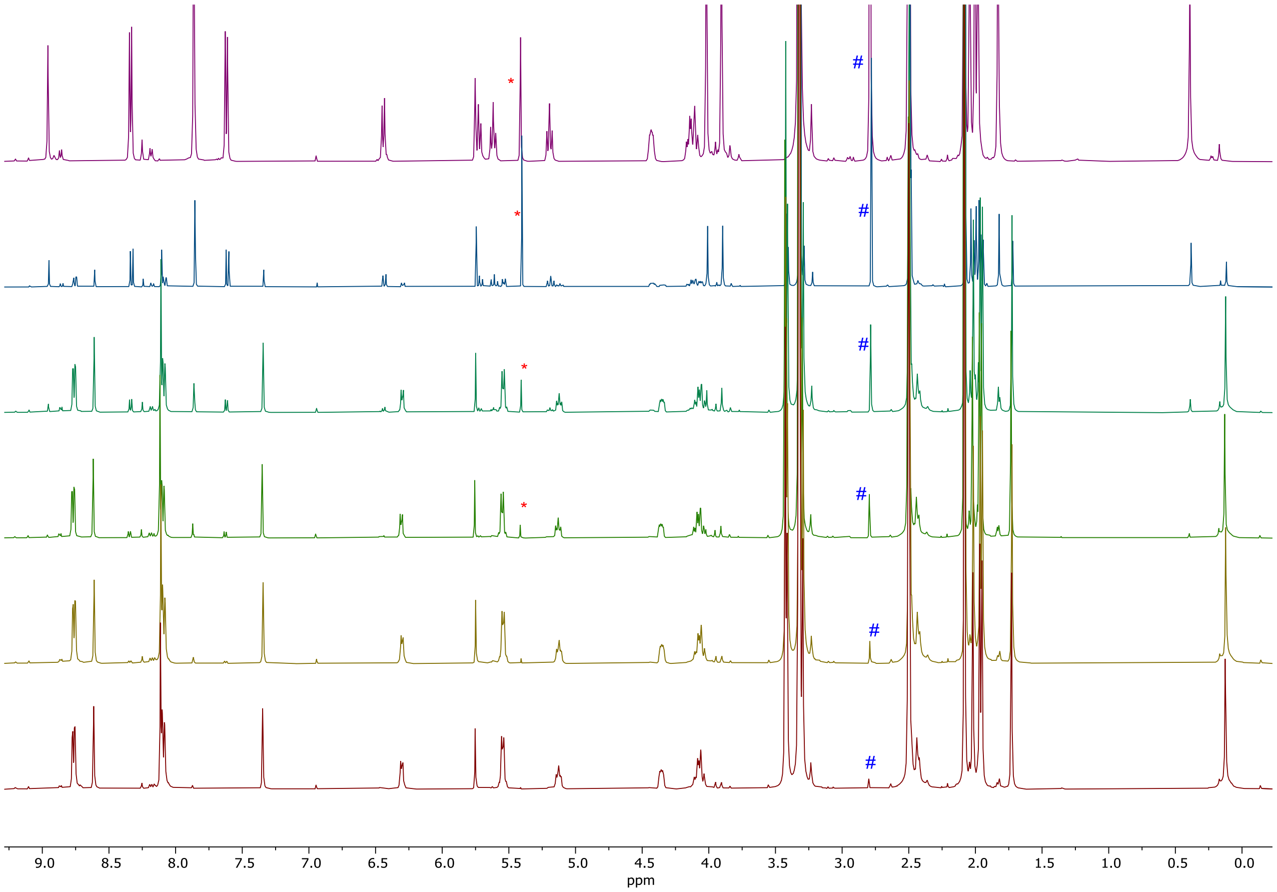


**Figure S13** - $^{1}$H NMR spectra of **1-Me,Me** in DMSO-$d_6$ after dissolution (bottom), 30 min, 2.5 h, 6 h, 24 h and 6 d (top) (400 and 500 MHZ, 298K, DMSO-$d_6$).

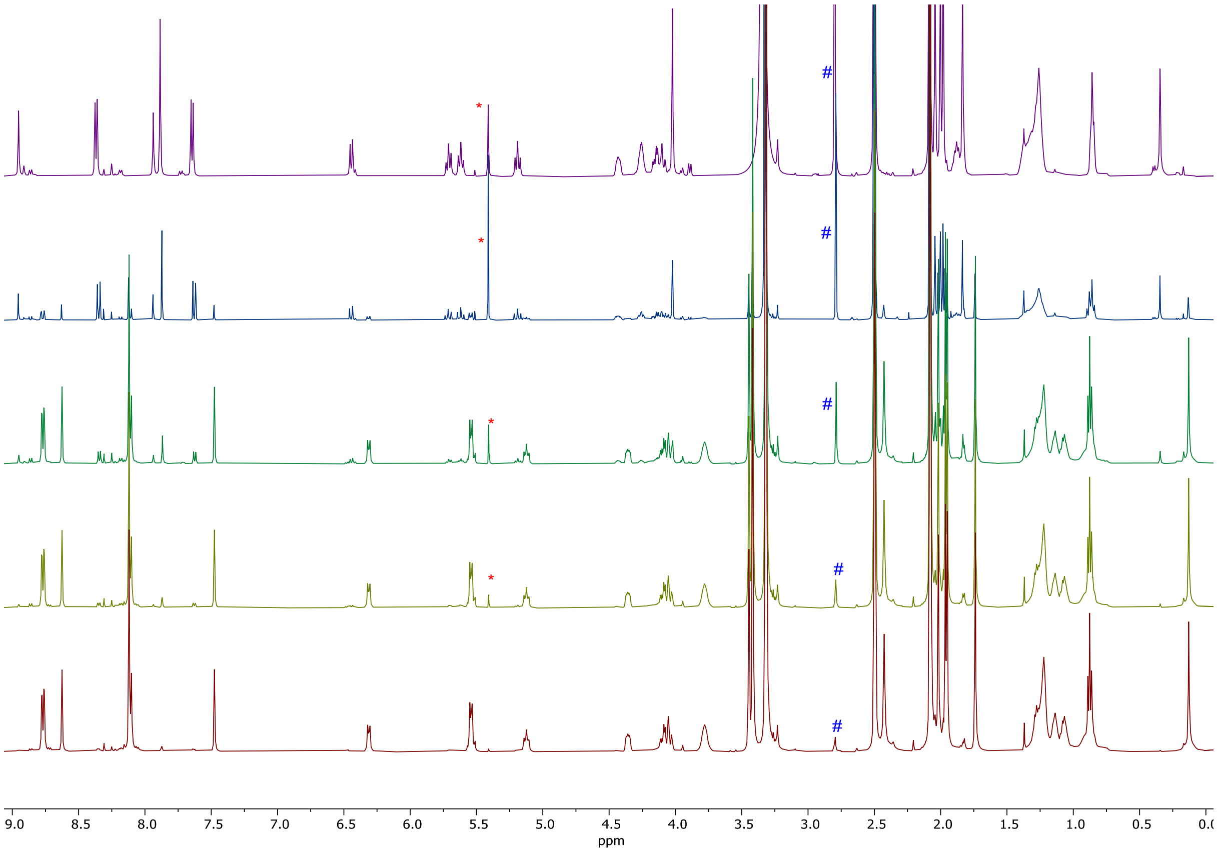


**Figure S14** - $^{1}$H NMR spectra of **1-Me,Oct** in DMSO-$d_6$ after 30 min (bottom), 2.5 h, 6 h, 24 h and 6 d (top) (400 and 500 MHZ, 298 K, DMSO-$d_6$).

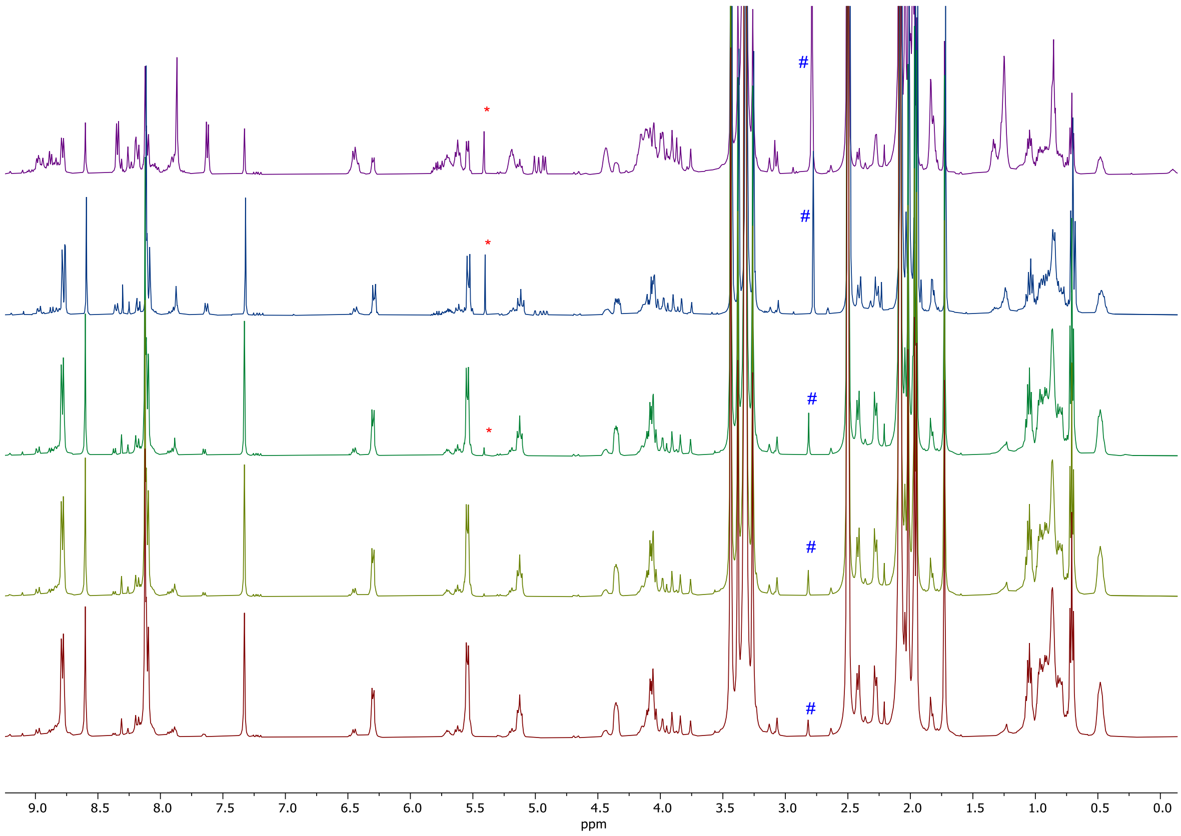


**Figure S15** - $^{1}$H NMR spectra of **1-Oct,Me** in DMSO-$d_6$ after 30 min (bottom), 2.5 h, 6 h, 24 h and 6 d (top) (400 and 500 MHZ, 298 K, DMSO-$d_6$).

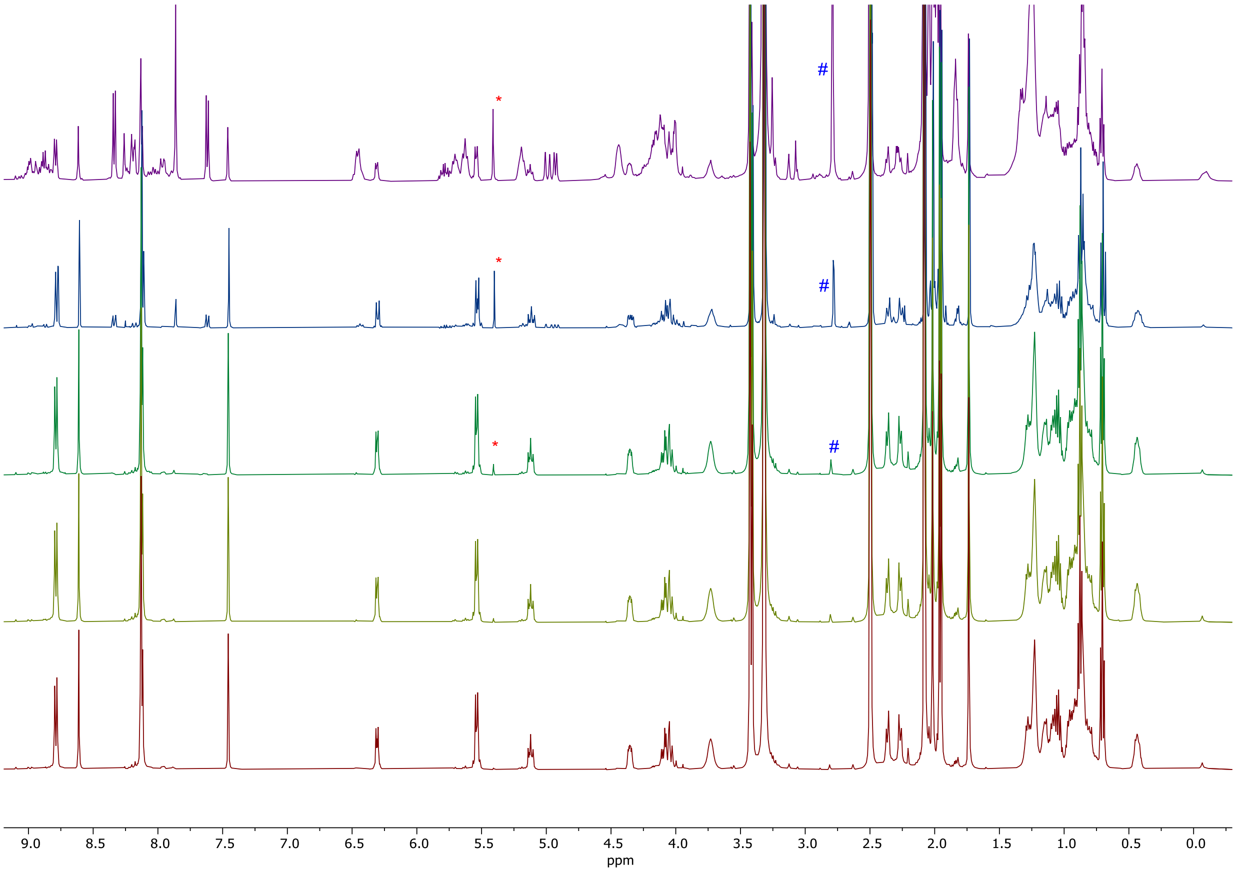


**Figure S16** - $^{1}$H NMR spectra of **1-Oct,Oct** in DMSO-$d_6$ after 30 min (bottom), 2.5 h, 6 h, 24 h and 6 d (top) (400 and 500 MHZ, 298 K, DMSO-$d_6$).

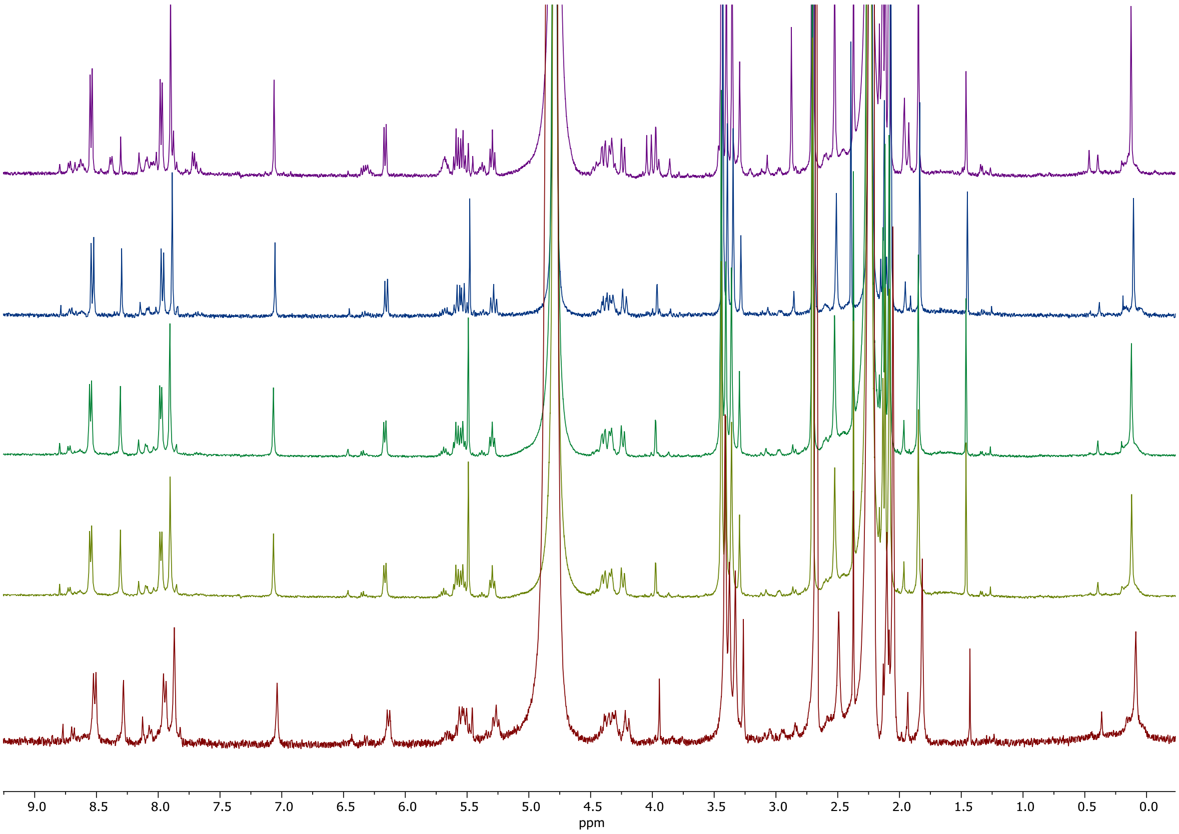


**Figure S17** - $^{1}$H NMR spectra of **1-Me,Me** in $D_2O$:DMSO-$d_6$ 10:1 after dissolution (bottom), 2.5 h, 6 h, 24 h and 6 d (top) (400 and 500 MHZ, 298K, $D_2O$).

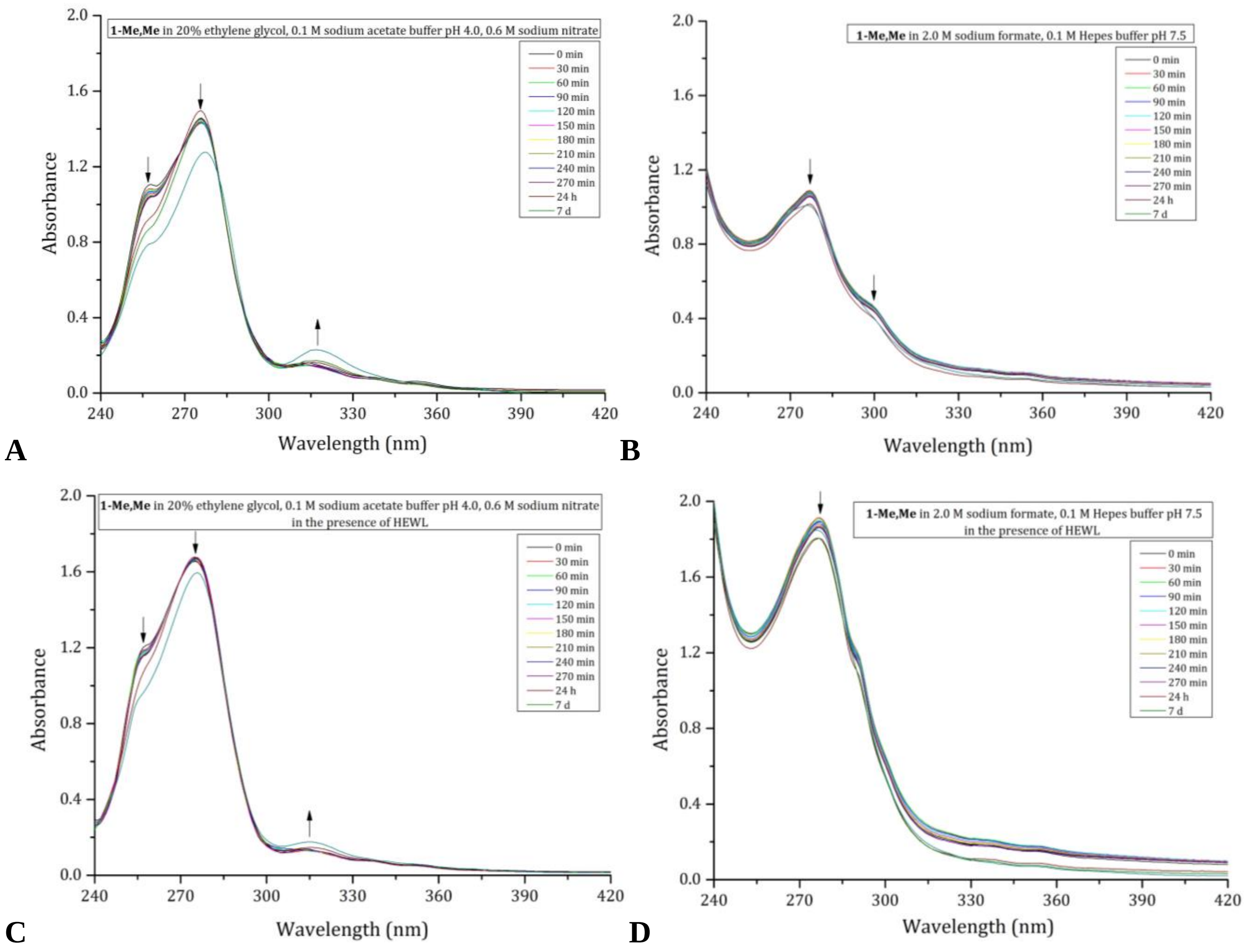


**Figure S18 -** Time course UV–vis spectra of 50 μM **1-Me,Me** in 20% ethylene glycol, 0.1 M sodium acetate buffer pH 4.0 and 0.6 M sodium nitrate (panels A and C), and 2.0 M sodium formate, 0.1 M Hepes buffer pH 7.5 (panels B and D) in the absence (panels A and B) and in the presence of HEWL (panels C and D) in a protein to metal molar ratio of 1:3.

**Table S2** - Data collection and refinement statistics.

| | Structure A | Structure B |
|---|---|---|
| ***Crystallization condition*** | in 20% ethylene glycol, 0.1 M sodium acetate buffer at pH 4.0, 0.6 M sodium nitrate | 2.0 M sodium formate, 0.1 M Hepes pH 7.5 |
| ***PDB code*** | 8BOY | 8BOV |
| ***Data collection*** | | |
| Space group | $P4_3\ 2_1\ 2$ | $P4_3\ 2_1\ 2$ |
| a (Å) | 77.50 | 78.58 |
| b (Å) | 77.50 | 78.58 |
| c (Å) | 37.58 | 37.05 |
| α/β/γ (°) | 90.0/90.0/90.0 | 90.0/90.0/90.0 |
| Molecules for asymmetric unit | 1 | 1 |
| Resolution range (Å) | 54.80-1.33 (1.35-1.33) | 55.56 - 1.25 (1.27-1.25) |
| Observations | 352268 (17028) | 412718 (19397) |
| Unique reflections | 27087 (1342) | 32587 (1623) |
| Completeness (%) | 100.0 (100.0) | 99.9 (100.0) |
| Redundancy | 13.0 (12.7) | 12.7 (12.0) |
| Rmerge (%) | 0.038 (1.251) | 0.046 (1.069) |
| Average I/σ(I) | 30.3 (2.2) | 22.9 (2.3) |
| $CC_{1/2}$ | 1.000 (0.812) | 0.999 (0.854) |
| Anom. completeness (%) | 100.0 (100.0) | 100.0 (100.0) |
| Anom. Multiplicity | 7.0 (6.6) | 6.7 (6.2) |
| ***Refinement*** | | |
| Resolution (Å) | 54.80-1.33 | 55.56 - 1.25 |
| N° reflections | 25524 | 31026 |
| N° reflections in working set | 1842 | 2229 |
| Rfactor/Rfree | 0.187/0.219 | 0.184/0.218 |
| N° non-H atoms in the refinement | 1253 | 1256 |
| Rmsd bonds (Å) | 0.013 | 0.016 |
| Rmsd angles (°) | 1.763 | 2.250 |

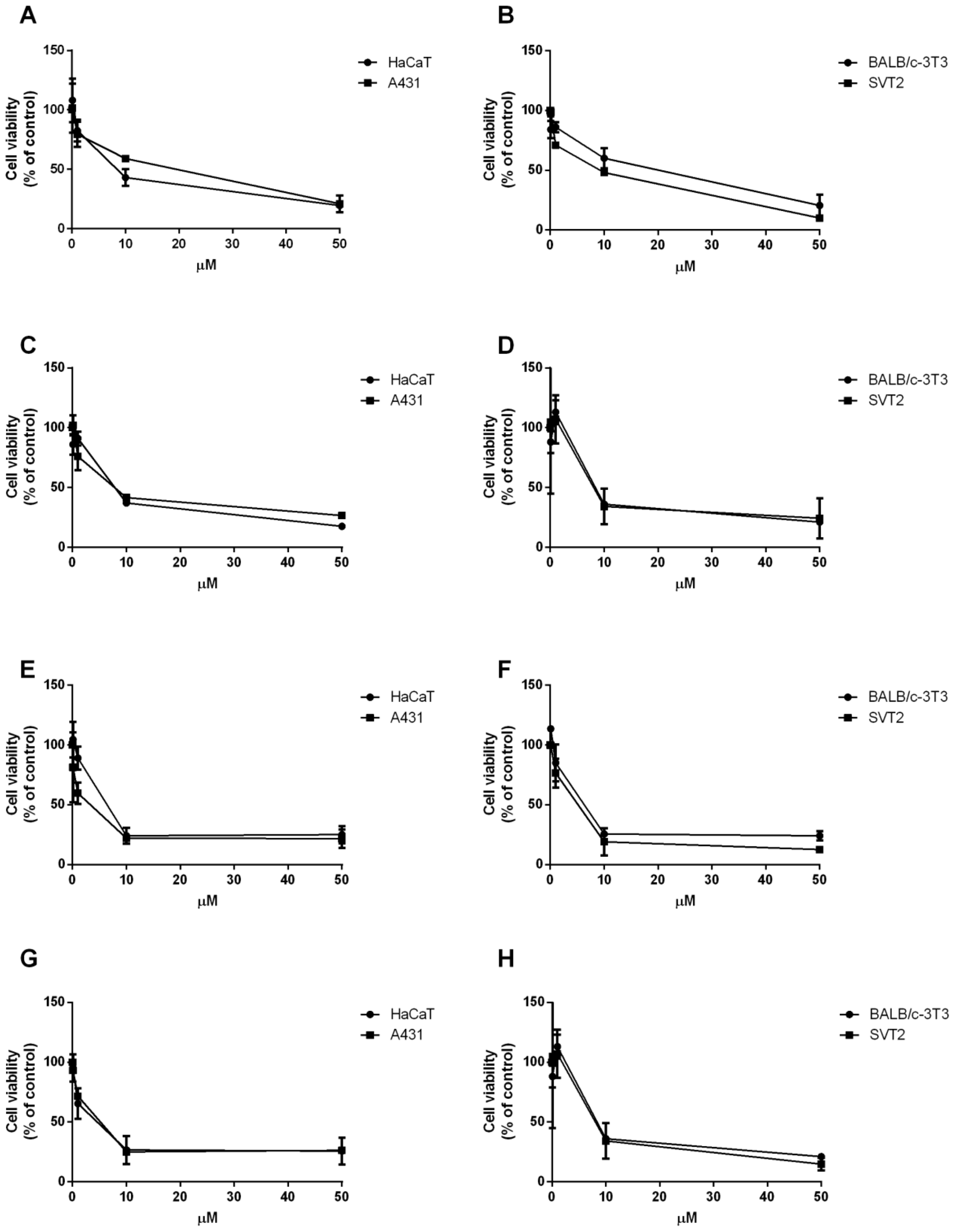


**Figure S19** - Effect of complexes on cell viability. Dose-response curve of cells incubated for 48 h in the presence of increasing concentration (0.1 – 50 μM) of each complex. **A**, **C**, **E**, **G**: HaCaT cells (black circles) and A431 cells (black squares); **B**, **D**, **F**, **H**: BALB/C-3T3 cells (black circles) and SVT2 cells (black squares). Cells were incubated with **1-Me,Me** (**A, B**); **1-Oct,Me** (**C, D**); **1-Me,Oct** (**E, F**); **1-Oct,Oct** (**G, H**). Cell viability was assessed by the MTT assay and expressed as described in Materials and Methods section. Values are given as means ± SD (n ≥ 3).

1)

$^{1}$H NMR (500 MHz, $CDCl_3$) δ 10.17 (s, 1H), 8.71 (s, 1H), 7.72 (s, 1H), 6.02 (d, *J* = 9.3 Hz, 1H), 5.57 (t, *J* = 9.4 Hz, 1H), 5.46 (t, *J* = 9.5 Hz, 1H), 5.31 (t, *J* = 9.8 Hz, 1H), 4.41 – 4.30 (m, 3H), 4.22 (s, 3H), 4.19 (dd, *J* = 12.5 Hz, *J* z= 1.8 Hz, 1H), 4.15 – 4.08 (m, 1H), 2.09 (s, 3H), 2.07 (s, 3H), 2.04 (s, 3H), 2.02 – 1.93 (m, 2H), 1.90 (s, 3H), 1.47 – 1.17 (m, 10H), 0.95 – 0.77 (m, 3H).
